# Meteorological effects of the solar eclipse of 20 March 2015: analysis of UK Met Office automatic weather station data and comparison with automatic weather station data from the Faroes and Iceland

**DOI:** 10.1098/rsta.2015.0212

**Published:** 2016-09-28

**Authors:** Edward Hanna, John Penman, Trausti Jónsson, Grant R. Bigg, Halldór Björnsson, Sølvi Sjúrðarson, Mads A. Hansen, John Cappelen, Robert G. Bryant

**Affiliations:** 1 Department of Geography, University of Sheffield, Sheffield S10 2TN, UK; 2 Met Office, Edinburgh EH11 3XQ, UK; 3 Icelandic Met Office, 108 Reykjavik, Iceland; 4 Deildin fyri infrakervi/Infrastructure Department, Landsverk, FO-110 Tórshavn, Faroes; 5 Danish Meteorological Institute, DK-2100 Copenhagen Ø, Denmark

**Keywords:** meteorology, pressure, solar eclipse, temperature, wind

## Abstract

Here, we analyse high-frequency (1 min) surface air temperature, mean sea-level pressure (MSLP), wind speed and direction and cloud-cover data acquired during the solar eclipse of 20 March 2015 from 76 UK Met Office weather stations, and compare the results with those from 30 weather stations in the Faroe Islands and 148 stations in Iceland. There was a statistically significant mean UK temperature drop of 0.83±0.63°C, which occurred over 39 min on average, and the minimum temperature lagged the peak of the eclipse by about 10 min. For a subset of 14 (16) relatively clear (cloudy) stations, the mean temperature drop was 0.91±0.78 (0.31±0.40)°C but the mean temperature drops for relatively calm and windy stations were almost identical. Mean wind speed dropped significantly by 9% on average during the first half of the eclipse. There was no discernible effect of the eclipse on the wind-direction or MSLP time series, and therefore we can discount any localized eclipse cyclone effect over Britain during this event. Similar changes in air temperature and wind speed are observed for Iceland, where conditions were generally clearer, but here too there was no evidence of an eclipse cyclone; in the Faroes, there was a much more muted meteorological signature.

This article is part of the themed issue ‘Atmospheric effects of solar eclipses stimulated by the 2015 UK eclipse’.

## 1. Introduction

**Figure 1.**
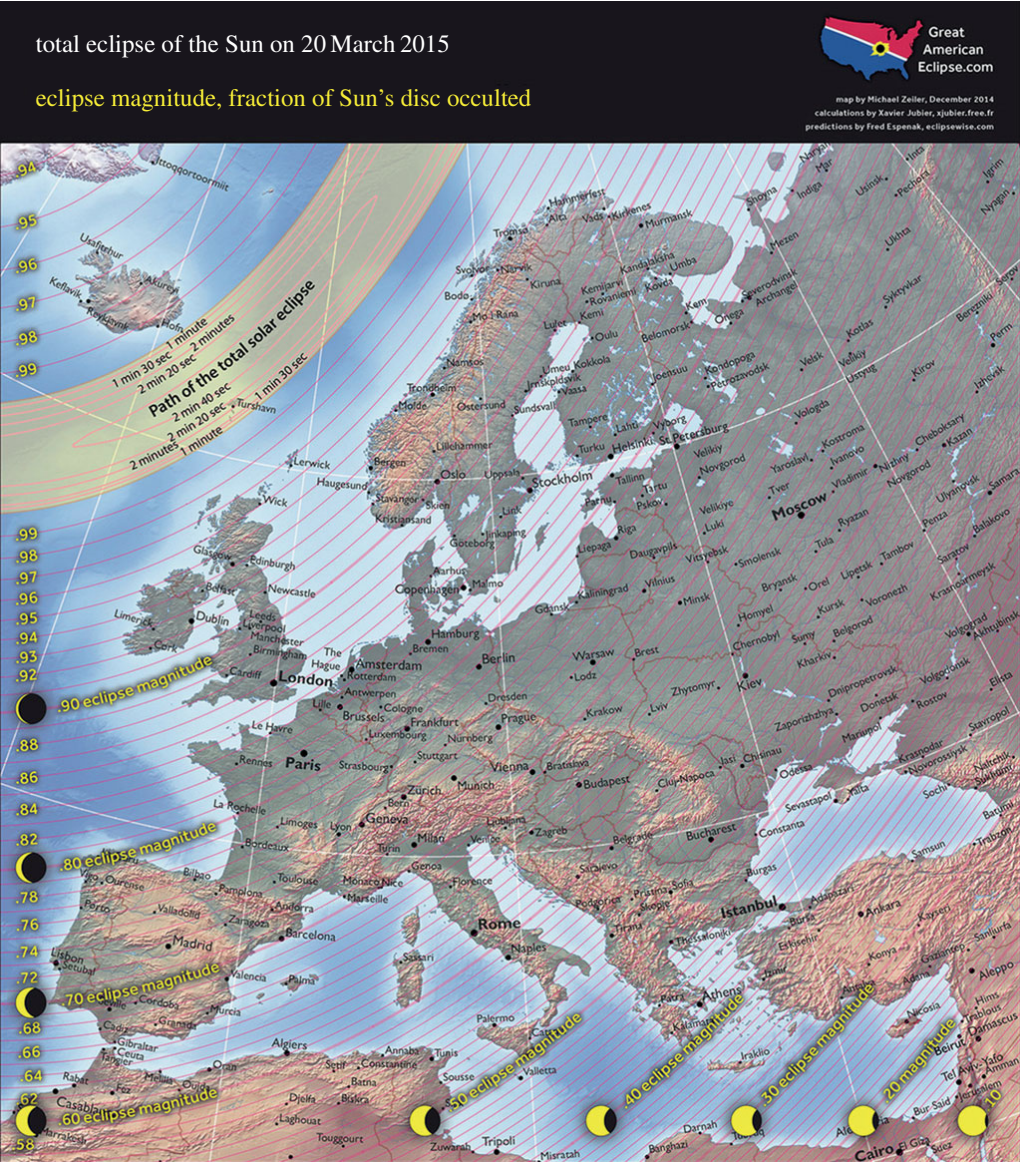
Path of the solar eclipse of 20 March 2015, showing fractional obscuration of the Sun at maximum eclipse. Image courtesy of http://www.greatamericaneclipse.com; copyright Michael Zeiler and reproduced with permission. (Online version in colour.)

On 20 March 2015, a very large partial solar eclipse occurred over the British Isles, with between about 85% and 97% of the solar disc obscured by the Sun (e.g. about 87% in London, 89% in Birmingham and 94% in Edinburgh). The eclipse was total in a swath several hundred kilometres north of the UK, extending south of Greenland and Iceland, across the Faroes and through to Svalbard (figure 1), and marked the last total solar eclipse visible from anywhere across Europe until 2026 (e.g. http://www.solareclipse2015.org.uk/). This was also the largest partial eclipse seen over Britain since the 11 August 1999 solar eclipse, with the previous event being total in most of Cornwall and parts of the Channel Islands and Devon [1]. A previous study examined the meteorological signature of the 1999 eclipse based on a network of about 80 amateur and professional weather stations across Britain [1]. Here, we repeat the exercise for the recent eclipse based on a similar number of well-distributed Met Office Meteorological Monitoring System (MMS) stations reporting data of surface air temperature, wind speed and direction, mean sea-level pressure (MSLP) and cloud cover every minute. Relatively few studies of this nature have been undertaken based on a dense network of meteorological stations, due to the relative rarity of solar eclipses taking place across suitably instrumented regions during modern times. However, previous studies, usually based on one to a few recording sites rather than dense station networks, report surface air temperature dips during solar eclipses of approximately 3°C (Paraguay [2]), 1.7–2.1°C (Germany [3]), 0.3–1.5°C (Svalbard [4]), 1.2–4°C (India [5–7]), up to 2.5°C (Korea [8]), 0.7–3.9°C (Greece [9,10]), 3.0°C (Antarctica [11]) and 3°C (Florida, USA [12]). Several workers also present evidence of an eclipse-related decrease in near-surface wind speed that can be attributed to more stable conditions in a cooler boundary layer (e.g. [2–4,6,9,10]). The improvements of the present study on Hanna [1] are a self-consistent database reporting measurements at a high time frequency that was only available for a subset of the stations during the previous event, and a comparison with dense networks of modern automatic weather stations (AWSs) from Iceland (just to the other side of the totality track) and the Faroe Islands (which fell under the path of totality).

The 20 March 2015 eclipse began (time of first contact) at around 0818 UTC in the Scilly Isles, 0825 in London and Birmingham, 0830 in Newcastle and Edinburgh and 0839 in the Shetland Islands; the time of maximum eclipse was 0923 at Scilly, 0931 in London and Birmingham, 0935 in Newcastle and Edinburgh and 0944 in Shetland; and the eclipse ended (fourth contact) at 1032 in Scilly, about 1040 in London and Birmingham, 1044 in Newcastle and Edinburgh and 1051 in Shetland [13] (all times in this paper are in UTC=GMT). The eclipse magnitude (fraction of the Sun’s disc occulted) at maximum eclipse ranged from 85% in Jersey to 87% in London, 88% in Scilly, 89% in Birmingham, 92% in Newcastle, 94% in Edinburgh, 96% in Wick and about 97% in Shetland and the Hebrides. The altitude of the Sun at maximum eclipse varied from approximately 22° in the Hebrides to approximately 29° in Kent. Corresponding measurements of solar radiation (affected both by astronomical/geometric considerations and by meteorological conditions, especially cloud cover) for 80 well-distributed UK sites reduced from a mean 134 W m^−2^ at 0900 to 27 W m^−2^ at 0930 (around mid-eclipse), then rose to 73 W m^−2^ at 1000 and 245 W m^−2^ at 1100.

**Figure 2.**
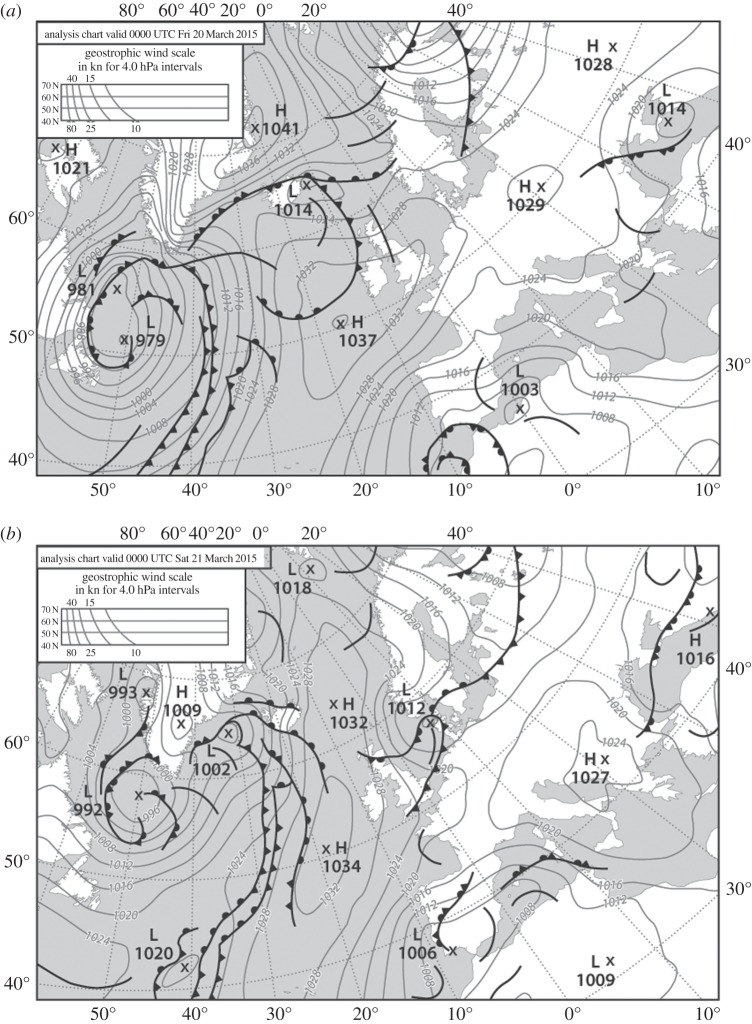
Met Office mean sea-level pressure chart for (*a*) 0000 UTC on 20 March 2015 and (*b*) 0000 UTC on 21 March 2015 (redrawn). Original charts obtained from the Wetterzentrale Topkarten website (http://www.wetterzentrale.de/).

**Figure 3.**
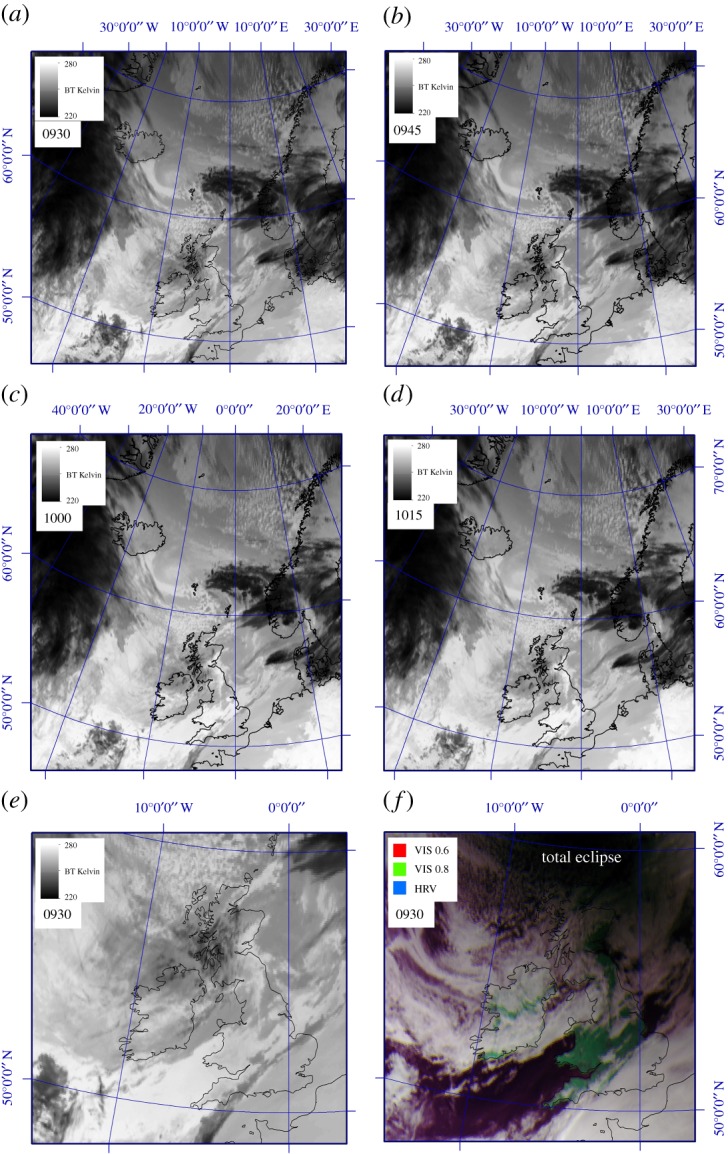
MSG-3 SEVIRI images collected on 20 March 2015 by the University of Sheffield receiving station. Images have been contrast-enhanced and all use a Polar Stereographic projection with a latitude/longitude grid overlain: (*a*–*d*) show variability in cloud-top temperatures (via the 10.8 μm SEVIRI waveband) for Northern Europe covering the period of the eclipse (from 0930 to 1015 UTC); (*e*) shows a detail from (*a*) allowing assessment of cloud cover and temperature variability over the UK during the eclipse period (0930 UTC); (*f*) is a multispectral false-colour rendition of (*e*) using optical wavelengths (*R*=0.6 μm, *G*=0.8 μm and *B*=HRV) obtained at 0930 UTC. Note the darkness within the total eclipse NW of the British Isles.

Meteorological conditions during the 20 March 2015 eclipse were largely settled, with high pressure over the British Isles (figure 2) but with decaying fronts and a maritime airflow around the northern and eastern edges of the high giving generally rather cloudy conditions (figure 3). Also of note is the tighter pressure gradient over the north of the UK, indicating breezier conditions. Multispectral satellite imagery shows the thickest/brightest cloud over southeast England and northern parts of both England and Scotland, while parts of southwest England, the Midlands and eastern Scotland were relatively clear during the eclipse (figure 3).

Because this eclipse occurred earlier in the morning and in spring rather than summer, in contrast with the 1999 event, compounded by fairly widespread cloud cover and breezy conditions in the north of the UK plus a dominant cool west to northwest airstream (figure 2), it was expected that eclipse-related temperature drops would be relatively muted for the 2015 event. With such a dense network of well-calibrated weather stations, however, it was feasible to search for evidence of the ‘eclipse cyclone’: a localized low-pressure circulation feature purported to arise from temporary cooling along the eclipse track and for which some evidence was found for the 1999 eclipse [14].

Section 2 of this study gives a detailed analysis of the Met Office weather station data for the UK, while §3 examines the meteorological response observed in the Faroe Islands (where the 2015 eclipse was total) and Iceland (where the eclipse’s magnitude ranged from 96% to more than 99%, e.g. approximately 97.5% in Reykjavik).

## 2. Automatic weather station (AWS) set-ups and specifications

For the MMS (UK) stations (electronic supplementary material, figure S1), dry-bulb temperature is measured using a four-wire electrical resistance thermometer. These are manufactured to the specifications determined by the Met Office and tested in accordance with BS EN 60751. They conform to ‘tolerance class A’. On deployment, the resolution is 0.1°C and the acceptable difference is less than ±0.2°C when checked against a reference (Inspectors) thermometer traceable to the National Physical Laboratory (NPL). Most sites use a standard Met Office Stevenson screen with no artificial ventilation or aspiration, although this is generally a modern plastic equivalent of the old traditional wooden Stevenson screen. For measuring wind speed and direction, the MMS stations use the Vector Instruments A100 L and W200P/L models, respectively. Solar radiation data were also obtained for a different but partly overlapping network of UK sites. UK Met Office radiation measurements are made by a Kipp & Zonen CM11 or CMP11 secondary standard instrument.

Electronic supplementary material, figure S2, shows the networks of Faroes AWSs run by Landsverk and the Danish Meteorological Institute (DMI). Landsverk runs a network of 26 AWSs gathering meteorological data every 10 min. Meteorological data are also available for six DMI Faroes stations, although two of these only report every 3 h and so are not considered here; the remaining four stations report either hourly (in three cases) or every 10 min (for station 6010; electronic supplementary material, figure S2). Most of the Faroes (Landsverk) AWSs are placed beside roads and either have the sloping side of a mountain on one side or are placed in a mountain pass. These AWSs use the Vaisala HMP155D thermo-/hygrometer, which is a 1/3 class B DIN platinum resistor element, with a DTR13 Radiation Shield to protect the sensors from solar radiation and rain, and there is no kind of forced ventilation. The DMI stations normally also use the Vaisala HMP155 thermo-/hygrometer, and this is coupled with another passive shield: the RM Young Multi-Plate Radiation Shield model 41003.

The Icelandic Met Office (IMO) AWS stations (electronic supplementary material, figure S3) use a Logan platinum-based sensor in a Young Multi-Plate Radiation Shield. Factory specifications list wind-speed-dependent radiation errors at 1080 W m^−2^ as 0.4°C at 3 m s^−1^, 0.7°C at 2 m s^−1^ and 1.5°C at 1 m s^−1^. Although most sites are standard, a few stations monitoring conditions in/near snow-support structures on steep avalanche-prone slopes are in operation in a non-standard solar radiation environment and must be considered to be atypical (station IDs 2640, 2641, 4181, 5992). Consideration was given to these stations in this paper and a comparison made with nearby standard stations; however, a detailed analysis is beyond the scope of this paper.

As is still fairly standard, none of these networks use any kind of forced ventilation on the radiation shields of their thermometers, which is a point we consider later in §5. Electronic supplementary material, figure S4, shows example AWS set-ups for the UK, Faroe Islands and Iceland.

## 3. Analysis of UK Meteorological Monitoring System data

Owing to demands of data analysis, MMS data were chosen for a set of 76 well-distributed sites across the UK (electronic supplementary material, figure S1). Here, we focus on a detailed analysis of the MMS data between 0600 and 1200 on 20 March 2015, which encompasses the whole of the eclipse period and about 90 min before and afterwards; this allows us to distinguish eclipse-related meteorological effects from the normal diurnal heating–cooling cycle. We present summaries of surface air temperature, wind speed and direction, MSLP and cloud-cover data taken every 15 min from 0830 to 1030 (electronic supplementary material, tables S1–S5), where the first and last times approximate the start and end of the eclipse and 0930 the eclipse peak. Table 1 presents an analysis of the highest temperature achieved after 0800 but before mid-eclipse, the lowest temperature attained during the eclipse period (typically around or just after mid-eclipse), and the time taken for the temperature drop. These are the actual temperature changes and they have not been corrected for the effect of the diurnal cycle (which often means that temperatures rise strongly at this time in the morning in March), and so tend to be conservative estimates of the full effect of the eclipse in suppressing temperatures compared with what they would otherwise have been.

**Table 1.** Highest and lowest temperatures, with their times (UTC), temperature drop and time taken, during the eclipse period on 20 March 2015.

station	high temp (°C), time	low temp (°C), time	drop (°C), time taken (min)
3/Fair Isle	7.2, 0903–0905	6.4, 0932/0934–0946	0.8, 27
9/Lerwick	6.8, 0855–0859	6.0, 0913/0914	0.8, 14
12/Baltasound	7.2, 0908–0915	6.4, 0942–0954	0.8, 27
23/Kirkwall	7.5, 0852–0900	6.8, 0941/0942 and 0953–0958	0.7, 41
32/Wick	7.1, 0912–0920	6.1, 0946–0952	1.0, 26
44/Altnaharra	7.7, 0855–0857	6.4, 0939–0958	1.3, 42
54/Stornoway	8.2, 0830–0842	7.9, 0903–0925	0.3, 21
67/Loch Glascarnoch	5.7, 0902–0903	4.8, 0949–1003	0.9, 46
113/Aviemore	8.6, 0843–0845 and 0904/0905	6.7, 1001	1.9, 56
132/Kinloss	8.1, 0900–0902	7.0, 0955–1006	1.1, 53
137/Lossiemouth	8.0, 0908–0917	7.1, 0945–1004	0.9, 28
150/Aboyne	11.4, 0837–0839	9.3, 0950–0953	2.1, 71
161/Dyce	10.4, 0914–0922	8.1, 1000–1011	2.3, 38
177/Inverbervie	8.8, 0922–0927	7.9, 0944–1002	0.9, 17
235/Leuchars	9.7, 0854–0900	8.3, 0935–0942	1.4, 35
268/Charterhall	9.1, 0907	7.4, 0941–0951	1.7, 34
315/Boulmer	10.8, 0850/0851	9.2, 0941–0948	1.6, 50
326/Durham	9.8, 0902–0903	8.8, 0945–0954	1.0, 42
346/Linton on Ouse	9.0, 0901–0902	8.2, 0932–0934 and 0947–1006	0.8, 30
360/Scarborough	7.7, 0849	5.4, 0940–0949	2.3, 51
384/Waddington	5.5, 0910	4.9, 0937–0946	0.6, 27
386/Cranwell	5.7, 0905	4.5, 0942–0945	1.2, 27
393/Coningsby	5.2, 0909–0917	4.5, 0939–0946	0.7, 22
409/Marham	4.7, 0855–0858	4.3, 0934	0.4, 36
421/Weybourne	5.5, 0914	4.9, 0935–0948	0.6, 21
426/Cromer	4.7, 0854	4.2, 0922	0.5, 28
471/Rothamsted	3.9, 0826	3.5, 0942	0.4, 76
525/Sheffield	9.1, 0839–0841	8.9, 0847–0901	0.2, 6
578/Northampton, Moulton	5.1, 0839–0849	4.2, 0940	0.9, 51
605/Brize Norton	6.0, 0859	5.4, 0929–0947	0.6, 30
613/Benson	4.5, 0900–0904	4.3, 0926–0946	0.2, 22
622/Keele	6.1, 0904–0911	5.0, 0946	1.1, 35
643/Shawbury	5.9, 0814–0847	4.4, 0941	1.5, 54
657/Pershore	5.7, 0907/0908	5.4, 0932–0943	0.3, 24
676/Filton	4.4, 0848–0915	4.2, 0931–0934	0.2, 16
697/London, St. James’s Park	5.5, 0843–0844	5.1, 0932–0939	0.4, 47
708/Heathrow	5.0, 0844–0900	4.7, 0913–0915 and 0924–0930	0.3, 24
709/Northolt	4.6, 0840–0841 and 0908–0911	4.4, 0931–0954	0.2, 20
723/Kew Gardens	5.2, 0856	4.8, 0924–0940	0.4, 28
775/Manston	5.0, 0831	4.3, 0931	0.7, 60
795/Shoreham	5.2, 0838–0839	4.6, 0925–0927	0.6, 46
811/Herstmonceux	4.9, 0839, 0844–0847	4.5, 0935–0939	0.4, 48
830/Reading	4.5, 0850–0913	4.3, 0920–0955	0.2, 7
842/Hurn	5.4, 0902	4.7, 0922–0934	0.7, 20
862/Odiham	4.0, 0911	3.8, 0921–0957	0.2, 10
876/Isle of Wight, St Catherine’s Point	5.6, 0826/0827	5.2, 0936/0937	0.4, 69
888/Larkhill	4.9, 0829	4.1, 0942–0948	0.8, 73
889/Boscombe	4.7, 0850/0851	4.3, 0909–0953	0.4, 18
1023/Eskdalemuir	7.1, 0834–0852, 0908–0911 and 0920	6.7, 0943–0949	0.4, 23
1046/Ronaldsway	9.2, 0830–0843	8.4, 0924–0933 and 0954–0956	0.8, 41
1060/Keswick	8.1, 0858–0905 and 0910/0911	7.7, 0931–0949	0.4, 20
1083/Shap	9.1, 0840–0843	7.4, 1006–1008	1.7, 83
1145/Valley	8.1, 0828–0832	7.3, 0928–0942 and 0946/0947	0.8, 56
1198/Aberporth	6.7, 0847–0850	2.9, 0950–0952	3.8, 60
1226/Pembrey Sands	5.5, 0901–0908	4.0, 0941/0942	1.5, 33
1255/Mumbles	6.6, 0832/0833	5.4, 0929–0934	1.2, 56
1302/Yeovilton	6.3, 0835–0846	6.1, 0905–0941, 0950, 0952–0954	0.2, 19
1319/Isle of Portland	5.4, 0827	4.9, 0923–0938, 0941	0.5, 56
1326/Swanage	5.1, 0803, 0859, 0930, etc.	4.8, 0908 and 4.9, 0934–0936	max 0.3, 9, etc.
1336/Plymouth, Mountbatten	5.4, 0846/0847	4.0, 0933–0934	1.4, 46
1346/Chivenor	3.6, 0908–0919	3.1, 0932–0935	0.5, 13
1386/Scilly	8.4, 0849–0855	7.8, 0925–0941	0.6, 30
1395/Camborne	6.6, 0854	6.1, 0928 and 0930	0.5, 34
1450/Aldergrove	7.9, 0855–0900	7.3, 0930–0942	0.6, 30
16588/Gravesend	5.0, 0903–0916	4.6, 0949	0.4, 33
17309/Crosby	7.8, 0849–0856	7.0, 0915–0921 and 0939–1013	0.8, 19
17314/Leeming	6.7, 0912/0913 and 0920	6.5, 0936–0939	0.2, 16
18903/South Uist Range	8.3, 0801–0806	6.9, 0956–1007	1.4, 110
18974/Tiree	8.6, 0848/0849	7.2, 0956–0958	1.4, 67
19187/Coleshill	6.4, 0903 and 0905	5.8, 0938–0949	0.6, 33
19260/Edinburgh	9.6, 0849–0853	8.7, 0940/0941 and 0944	0.9, 47
25727/Southampton	5.4, 0850/0851 and 0906	5.2, 0911–0920	0.2, 5
30620/Charlwood	4.5, 0827–0843	4.3, 0919–0950	0.2, 36
55827/Braemar	8.4, 0835–0837	7.5, 0946–0957	0.9, 71
56937/Giant’s Causeway	7.5, 0854–0911	7.3, 0923–0934 and 0945	0.2, 12
56958/Emley Moor	7.1, 0838–0858	6.0, 0938–0940	1.1, 40

**Figure 4.**
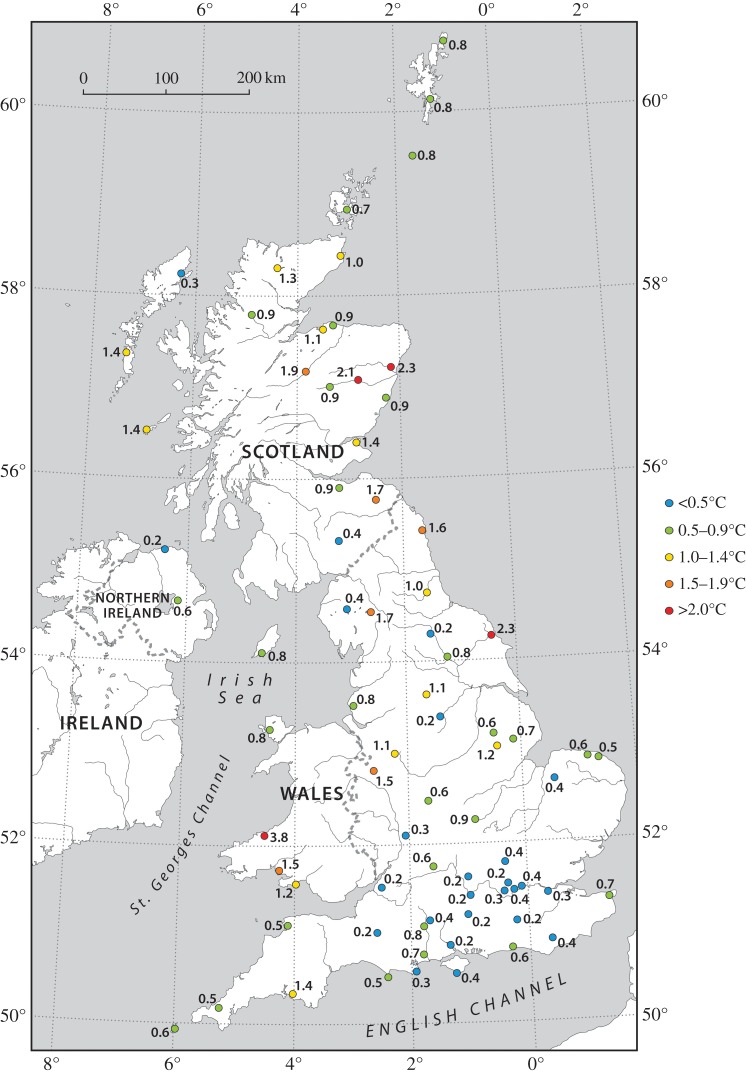
Surface air temperature drops during the eclipse based on 1 min MMS data (see also table 1 and figure 5).

**Figure 5.**
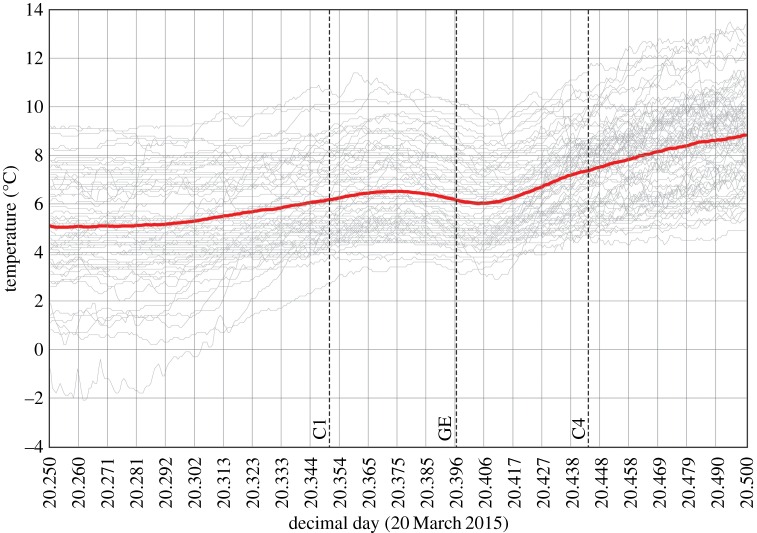
Surface air temperature at 76 weather stations across the UK between 0600 and 1200 UTC on20 March 2015 (figure 4; electronic supplementary material, figure S1), with the bold (red) line marking the mean of all stations. The vertical lines C1, GE and C4 mark the times of first contact, maximum eclipse and fourth contact. (Online version in colour.)

All sites show a discernible decrease in temperature during the eclipse but, as expected, this varies greatly (table 1; figures 4 and 5). The mean temperature decrease for all sites, based on slightly different time periods for stations, is 0.83±0.63°C (where ± denotes 1 s.d.). The sites of greatest cooling (up to 4°C) tended to be in eastern/central Scotland, parts of northern England and Wales, which tended to coincide with clear spots on the satellite images (figure 3), which is presumably due to greater radiational cooling at these sites (i.e. a stronger decline in solar radiation and/or larger loss of terrestrial radiation under clearer skies); conversely, the least cooling (as little as 0.2°C) tended to be at sites in southeast England, which had a band of thicker cloud cover during the eclipse (figure 3). However, based on regression analysis, nearly all the temperature declines at the 76 MMS stations are statistically significant (*p*≤0.05), with just three exceptions: Sheffield, Filton (Bristol) and London St. James’s Park.

Averaging the 1 min temperature data every 15 min at all sites (bottom row in electronic supplementary material, table S1) has the effect of smoothing the original temperature series because the times of peak high and low temperature vary between sites. The mean temperature of all the sites was 6.51°C at 0900 and 6.02°C at 0945, and looking at the more detailed 1 min data the temperature peak lasted from 0855 to 0902 and the low from 0941 to 0945, giving a mean time of 39 min taken for the temperature drop. This cooling of 0.49°C is more modest than the 0.83°C reported in the previous paragraph because there has inevitably been some smoothing of the original temperature data series during the averaging process. The peak temperature occurred approximately 30±17 min after first contact, and there was a shorter lag of approximately 10±14 min of the low temperature following greatest eclipse (± denotes standard deviation of the different times of high or low temperature for individual stations noted in table 1).

**Figure 6.**
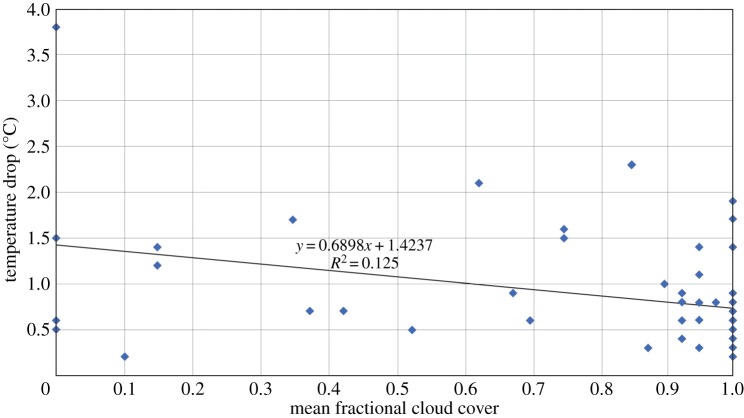
Surface air temperature drop during the eclipse versus mean cloud cover for UK stations in the first half of the eclipse (0830–0930). The data series are correlated at the *p*≤0.01 significance level. (Online version in colour.)

There is very little difference in the magnitude of the temperature decrease for subsets of relatively windy (more than or equal to 8 kn at 0930; *n*=23) and less windy (less than or equal to 5 kn; *n*=20) sites, with these subsets having almost identical temperature drops of 0.54±0.31°C and 0.55±0.75°C, respectively. There is also no relationship between wind speed at any of the 15 min time steps during the eclipse and the size of the temperature decline as defined in the previous paragraph. On the other hand, 14 sites having cloud cover of less than or equal to 5 oktas at all the 15 min time steps during the eclipse experienced a considerably greater mean temperature decline (0.91±0.78°C) than cloudy sites with consistently 8 oktas of cloud cover (0.31±0.40°C; *n*=16): this difference is statistically significant at the 95% confidence level using a Student’s *t*-test. There is also a weak but significant negative correlation between mean cloud cover during the first half of the eclipse and temperature drop (figure 6).

**Figure 7.**
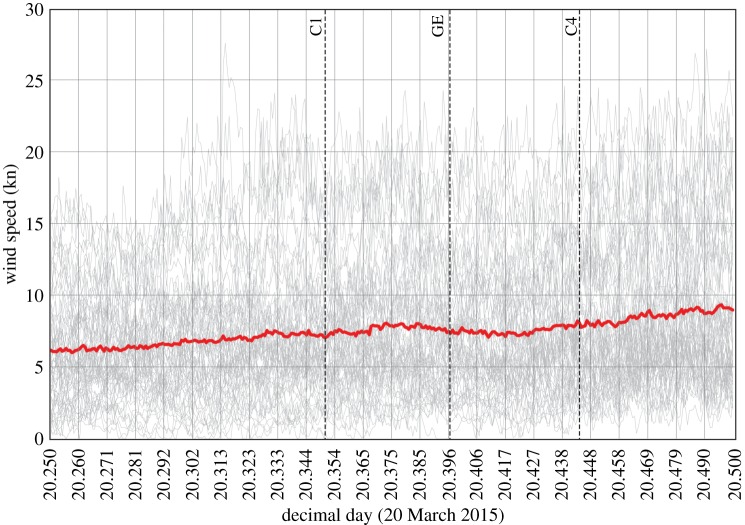
Wind speed at 63 weather stations across the UK between 0600 and 1200 UTC on 20 March 2015, withthe bold (red) line marking the mean of all stations. The vertical lines C1, GE and C4 mark the times of first contact, maximum eclipse and fourth contact. (Online version in colour.)

There is a discernible reduction of wind speed during the eclipse from 8.14 kn at 0857 to 7.38 kn at 0935, which is seen as a temporary reversal of the normal diurnal increase in wind speed at this time of the morning and/or synoptic changes (figure 7; bottom row of electronic supplementary material, table S2). However, while the decrease in wind is significant (*p*≤0.05) across all station data (when averaged), based on individual station data, this is only true for 22 out of 63 sites, whereas 15 sites show a significant increase during the 0900–0930 time period. Also, there is no significant relationship between the drop in temperature and the drop in wind speed. There is also evidence of cloud cover having cleared at a number of sites, including Wick, Charterhall, Coningsby, Shawbury, Camborne and Coleshill, as the eclipse progressed (electronic supplementary material, table S5), although, since the distribution of the sites shows no pattern and there is no correlation with temperature decreases, this may simply be part of a trend towards less cloud at these particular stations during the morning rather than a temporary drop driven by the eclipse. There is no discernible eclipse signature on either the wind-direction or MSLP time series, for either the mean or individual profiles (electronic supplementary material, figures S5 and S6). A map of MSLP values for peak eclipse (0930) simply reflects the general synoptic pressure gradient (figure 2) and shows absolutely no evidence of an eclipse cyclone either (electronic supplementary material, figure S7). A further analysis of MSLP changes during the first and second halves of the eclipse (not presented here in detail but based on data in electronic supplementary material, table S4) shows no systematic (total or regional) MSLP drops during the first half of the eclipse, or rises in the second half, that could be related to a possible eclipse cyclone.

## 4. Comparison of UK eclipse signature with Faroes and Iceland meteorological data

Here, we compare the results above with AWS data collected from the Faroes (where the eclipse was total, with first contact at approximately 0839, with two minutes of totality at approximately 0942 and fourth contact at approximately 1048 [13]) and Iceland (where the eclipse magnitude reached 96% to more than 99%, with times of start, end and greatest eclipse similar to the above). The locations of the two sets of stations are shown in electronic supplementary material, figures S2 and S3.

### (a) Faroes

Electronic supplementary material, tables S6–S8, summarize the usable DMI data from four sites for 0600–1200 on 20 March 2015. The mean temperature at 23 Landsverk stations (ignoring three with significant gaps, which would bias the mean) was 5.98°C at 0838, 6.07°C at 0938 and 6.24°C at 0948, with no discernible temperature drop during the eclipse. This remains the case when these stations are subdivided into ones above and below 100 m elevation (electronic supplementary material, figure S8), although even the higher-elevation stations (280 m for F12, 278 m for F49, 273 m for F33 and 245 m for F43) are near the coast and have a strong maritime influence. Also for the Faroes, there is no discernible wind-speed signature during the eclipse (electronic supplementary material, figure S9), neither is there any sign of a barometric pressure anomaly (which might be indicative of an eclipse cyclone) around mid-eclipse (electronic supplementary material, figure S10). Wind direction (not shown) was predominantly northwesterly and remained stable with no anomalous changes during and around the eclipse period. DMI stations 6011 Torshavn and 6012 Kirkja Fugloy cloud data show 90% cover at 0800, 0900, 1000 and 1100 during and immediately before/after the eclipse, and 6010 Vaga Floghavn cloud cover was 90% before and after the eclipse but did temporarily reduce to 25% at 0940, representing a partial clearance during mid-eclipse (electronic supplementary material, tables S6–S8). Station 6010 also shows a discernible reduction in temperature during the eclipse: from 6.8°C at 0850 to 5.7°C at 1020. Temperature drops of 0.6–1°C are also apparent around the time of the eclipse in several of the Landsverk temperature records (specifically F12, F22, F23, F35 and F41; electronic supplementary material, figure S8) but are insufficiently prevalent to translate into the Landsverk mean temperature profile. The six stations with discernible temperature drops (including 6010) are clustered mainly in the northwest of the Faroes archipelago but with one exception, F22 in the eastern part of the central north (electronic supplementary material, figure S2), and they are in the part of the islands where we have noted the partial cloud clearance. There is no obvious maritime exposure, elevation or topographic bias in these stations. We do not have sufficiently high-resolution satellite data to reliably discern local cloud-cover changes over the Faroes but the surface observations indicate that the northwest happened to lie in a relatively clear spot that allowed the temperature there to fall measurably around the time of totality.

### (b) Iceland

**Figure 8.**
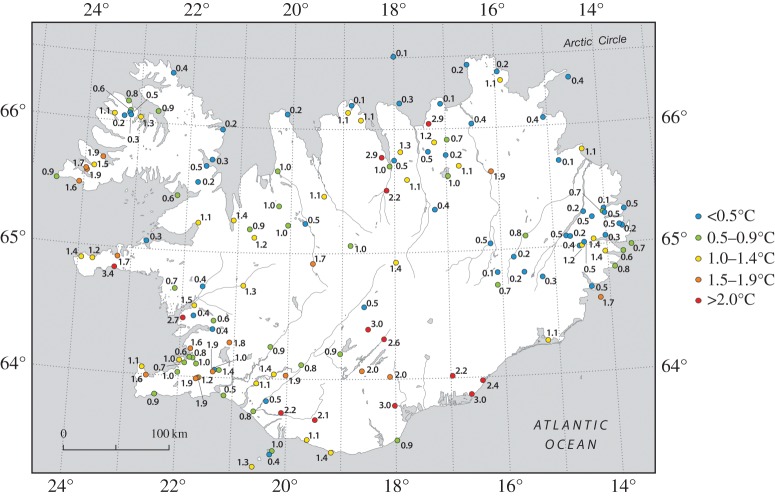
Surface air temperature reductions at Icelandic Met Office automatic weather stations during the 20 March 2015 solar eclipse.

**Figure 9.**
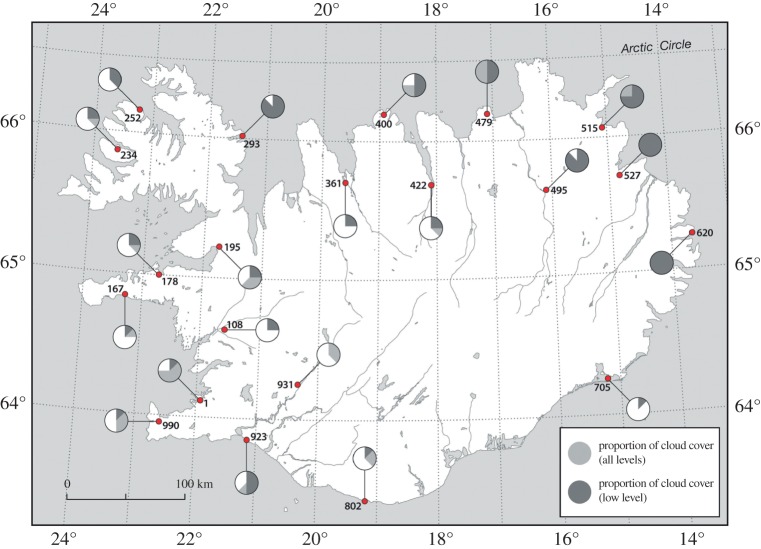
Cloud-cover conditions in Iceland at 0900 UTC on 20 March 2015, during the first half of the eclipse.

**Figure 10.**
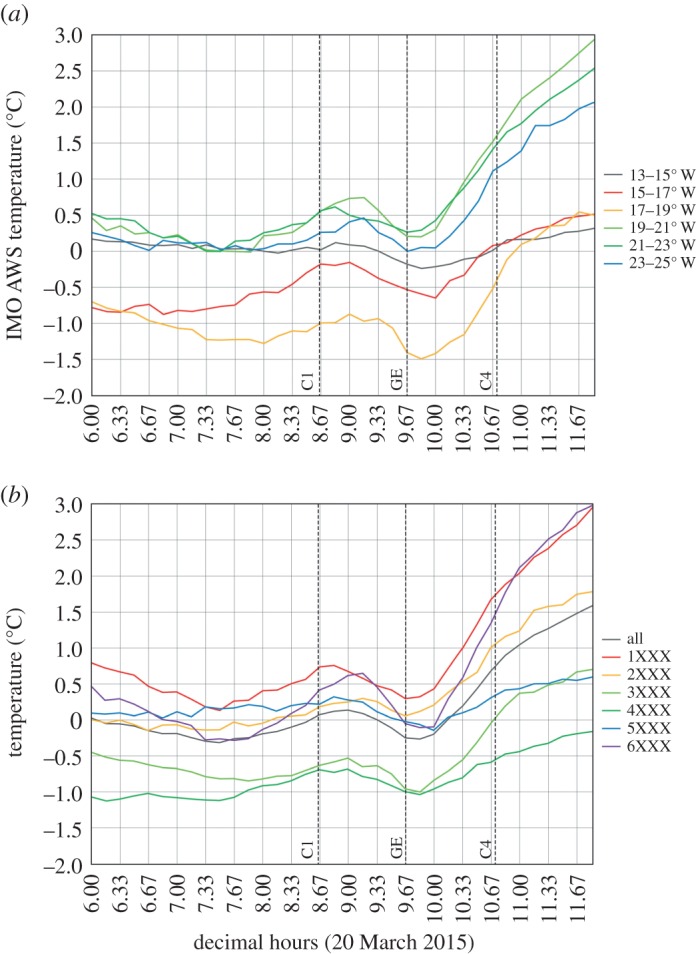
Mean surface air temperature response in Iceland during the 20 March 2015 eclipse: (*a*) by longitude; (*b*) by region (see the main text for full explanation). The vertical lines C1, GE and C4 mark the times of first contact, maximum eclipse and fourth contact.

The main AWS dataset of the IMO comprises 10 min reports of temperature together with the maximum temperature and minimum temperature during the preceding 10 min. We use the highest maximum temperature during 0830–0950 and the lowest minimum temperature during 0940–1100 to define the magnitude of temperature decrease at individual stations during the eclipse. These data are plotted in figure 8 and show the greatest declines generally in the south and west, and the smallest temperature drops in the north and east. In addition, cloud-cover data are available for a sparser network of synoptic stations and are plotted for 0900 (during the first half of the eclipse) on 20 March (figure 9). In contrast with the UK and Faroes, these indicate that moderately clear to partly cloudy conditions prevailed over much of the country—especially in the south and west (e.g. the eclipse was well seen from Reykjavik)—but it was rather cloudy in northeast Iceland. The temperature data anecdotally suggest a strong role of cloud cover in suppressing the eclipse-related temperature decline. This potential effect is explored more by binning the temperature data according to region and longitude range and graphically presenting the results (figure 10). Regions are defined as IMO AWS numbers suffixed with 1XXX…6XXX in the electronic supplementary material, figure S3. The eclipse magnitude effect was mainly longitudinal in Iceland (figure 1). The mean regional temperature drop determined from 10 min AWS data varies from 0.24±0.33°C for stations suffixed 2XXX (northwest Iceland; electronic supplementary material, figure S3) to 0.72±0.65°C for stations suffixed 6XXX (southern Iceland). For the latter region, conditions were relatively clear. The mean temperature drop by longitude band is: 0.36±0.31°C for 13–15° W (the cloudy east), 0.50±0.68°C for 15–17° W, 0.61±0.88°C for 17–19° W, 0.55±0.49°C for 19–21° W, 0.36±0.46°C for 21–23° W and 0.47±0.55°C for 23–25° W. For the lattermost two regions (westernmost stations), skies were partially clear; yet the mean temperature drop for the 21–23° W (encompassing Reykjavik but also many other stations) is no greater than in the cloudier east—this might be attributed, at least in part, to the eclipse magnitude being approximately 2% greater in the east and/or to the southwest of the country being more exposed to maritime conditions. Incidentally, at Reykjavik (where the magnitude of the eclipse peaked at between 97% and 98%) short-wave radiation declined from around 34 W m^−2^ at 0850 to just 2 W m^−2^ at 0940 before rising steeply thereafter, reaching approximately 60 W m^−2^ by 1035.

**Figure 11.**
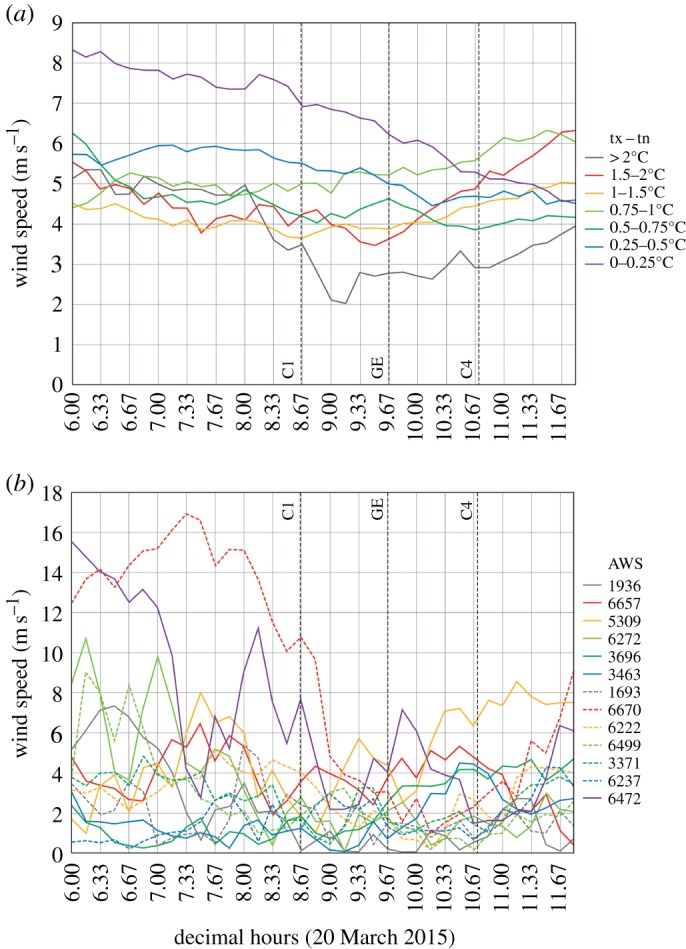
Wind speed at Icelandic automatic weather stations between 0600 and 1200 UTC on 20 March 2015, encompassing the solar eclipse period: (*a*) shows means of AWSs with different ranges of tx − tn temperature drops during the eclipse; (*b*) shows individual AWSs where the temperature drop exceeded 2.0°C. The vertical lines C1, GE and C4 mark the times of first contact, maximum eclipse and fourth contact.

There is also an apparent reduction in wind speed at Icelandic AWSs during the eclipse, as seen with the UK data, but this is superimposed on a trend of decreasing wind speed during the 6 h period centred on the eclipse. The wind reduction is large and obvious for stations where the decline in temperature exceeds 1.5°C and much less apparent for stations with more modest temperature decreases (figure 11): for example, for the 15 Icelandic stations where temperature declined by more than or equal to 2°C (less than 1°C) during the eclipse, mean wind speed fell from 4.96 (5.70) m s^−1^ at 0800 to 2.03 (5.35) m s^−1^ at 0910. Therefore, this drop in wind speed is probably related to the eclipse.

Finally, electronic supplementary material, figure S11, shows the MSLP at IMO AWS for 0930 UTC (around the time of mid-eclipse) and the MSLP changes during the first and second halves of the eclipse (0840–0940 and 0940–1040). Electronic supplementary material, figure S11*a*, does not show a cyclonic-type circulation pattern anywhere over Iceland—in fact, it shows an anticyclonic anomaly of approximately +1 hPa in central southern Iceland—and electronic supplementary material, figure S11*b*, does not show any systematic decrease in MSLP during the first half of the eclipse that might be related to a possible eclipse cyclone—indeed MSLP changes were generally more positive during the first half of the eclipse (mean change 0.33 hPa) compared with the second hour (mean change 0.06 hPa): this bias is especially evident in inland southern Iceland (electronic supplementary material, figure S11*b*).

## 5. Discussion and summary

Weather conditions over the British Isles during the 20 March 2015 eclipse were not optimal, due to fairly extensive cloud cover, but were also fairly stable under a ridge of high pressure and the cloud cover was patchy and broken in places. Therefore, there was a wide range of temperature drops experienced, from typically just a few tenths of a degree Celsius in southeast England to elsewhere locally in excess of 2°C. A previous comprehensive analysis of observational data acquired during the 11 August 1999 solar eclipse found mean surface air temperature drops ranging from 1.2°C in southwest England and 1.3°C in Wales and Scotland to 2.3°C in southeast England, with a few sites recording a temperature decline of 3°C or more [1]. As originally anticipated, these summertime values tend to be somewhat greater on the whole than the temperature drops experienced during the 20 March 2015 eclipse, although there is considerable overlap between the ranges of temperature drops experienced during the two eclipses. As we have seen above, where skies were clear (or relatively cloudless), temperature drops in excess of 2 or 3°C occurred at a few sites during the more recent event. Temperature lows occurred approximately 10 min after peak eclipse, which is broadly in line with previous meteorological studies (e.g. 10 to 15 min [15]; 15 min [9]; 20 min [6]). Also, we point out that temperature declines during the 20 March 2015 eclipse would probably have been considerably greater overall had skies been generally clearer or winds lighter and from a drier land area (e.g. easterly), and/or if a low inversion level allowed temperature changes to be distributed across a shallower depth.

Part of the measured fall in air temperature as the eclipse advanced might have been due to the effect of the reduction in solar radiation incident upon the thermometer screens themselves rather than to a change in actual air temperature. Readings made in naturally aspirated thermometer screens can be higher than the ambient air temperature due to the warming of the screens by solar radiation, especially at low wind speeds. For example, Hanna *et al*. [16] examined this effect in relation to new temperature records and warming trends accompanying the July 2012 record surface melting of the Greenland Ice Sheet. As a solar eclipse progresses and radiation falls, any spurious radiational heating effect will be lessened and the indicated temperature might fall slightly as a result. On the other hand, one might expect this effect to be counteracted by a general drop in wind speed towards maximum eclipse. Wind speeds occurring during the 20 March 2015 eclipse were typically about 3 to 4 m s^−1^, and—as we have seen—did reduce at quite a few of the sites around mid-eclipse. None of the national meteorological networks in this study use aspirated screens (which are much less prone to being affected by solar radiation), but the temperature drop at cloud-covered sites and auxiliary data available for a few weather stations outside these networks suggest that a significant part of the observed change in temperature was due to a real fall and recovery in the air temperature. For example, the lead author maintains a Davis Vantage Pro 2 AWS with a Fan Aspirated Radiation Shield (FARS) in his northeast-facing back garden, lakeside, suburban/semi-rural (town edge) site in Newark-on-Trent, Nottinghamshire, where skies were relatively clear and the Sun was visible for the duration of the eclipse (solar radiation initially peaked at 319 W m^−2^ at 0835, then fell to 80 W m^−2^ at 0935 around mid-eclipse, afterwards rising to 512 W m^−2^ at 1035). This station is part of the Climatological Observers Link, and data are logged every 5 min. The small plastic radiation shield (electronic supplementary material, figure S4*d*) is broadly comparable with similar designs used in the Faroes and Iceland AWS networks. During a calibration exercise in 2015, the Davis thermistor was found to agree within 0.2°C with a standard UKAS-calibrated reference thermometer over a temperature range of 5–35°C (*n*=81). On 20 March 2015, the 2 m air temperature peaked at 6.6°C at around 0900 before dropping to 6.2°C at around 1000 (about half an hour after mid-eclipse), then rising steeply to 8.2°C by 1030 and 12.2°C by 1140. The temperature drop of 0.4°C during the eclipse is not far off the 0.6°C drop measured at the relatively nearby (approx. 20 km to the northeast) MMS site of Waddington, although it is only a third of the 1.2°C drop observed for Cranwell, which is located a similar distance to the east (table 1). However, it is greater than the manufacturer’s specified solar radiation-induced error of 0.3°C for the FARS at solar noon, which, given a steady breeze and the early morning Sun fairly low in the sky, is likely to significantly overestimate the actual error with this shield during the eclipse. It is also worth reiterating that around the time of peak eclipse under relatively clear skies and a stable airmass the temperature would normally have been rising strongly, so the eclipse-induced temperature effect is very likely to be greater than these figures suggest.

Our results indicate that cloud cover is a much more important determinant than wind speed in controlling the surface air temperature response during this eclipse. Also, because there is no obvious north–south gradient in temperature decreases over the UK (figure 5), it is clear that local cloud-cover variations are far more influential than small (5–10%) variations in eclipse magnitude across the country in affecting surface cooling. This is in line with previous work, e.g. an analysis of three meteorological stations during the 2008 solar eclipse over Greece [9].

There was a significant (approx. 9%) reduction in mean UK wind speed as the eclipse progressed, which counteracted the natural tendency of wind to strengthen during this period due to diurnal/synoptic effects; this effect was previously found for the 1999 eclipse and reflects more stable boundary-layer conditions associated with the temporary cooling [1]. Cloud clearance during an eclipse is also something that has previously been observed [1], and again we present data showing that this may have happened for selected UK stations on 20 March 2015, although the evidence here is weaker and there is no clear geographical pattern. Our analysis of wind-direction and MSLP data from numerous UK and Icelandic sites discounts the possibility of the much-fabled eclipse cyclone having occurred during the recent event, although this is not to rule out such a phenomenon under more favourable meteorological conditions, perhaps, under conditions of strong solar heating in summer or during a lower-latitude eclipse over land.

The Iceland AWS data show a range of temperature drops from approximately 0.1 to 3.3°C during the eclipse, which are much in line with the results for the UK. The greatest cooling occurred in southern Iceland, especially inland, although a few locations in far northern Iceland also experienced temperature drops as great as 2.9°C. At this time of the year, mean temperatures at the Iceland stations were close to 0°C (with available energy potentially being used in snow and ice melt at some sites), and this, together with the lower solar elevation, should lead to a smaller temperature signature than in the UK. The redeeming factor was the generally lower cloud cover in Iceland, especially in the south and west, which meant that the temperature response ended up being similar to that observed in the UK. The lower time resolution of the Icelandic data, compared with the UK Met Office MMS data discussed above, does not permit us to analyse the peak eclipse low temperature time lag with comparable accuracy. However, the availability of maximum and minimum temperature records between the 10 min set observation times does make the comparison of temperature decreases methodologically comparable between the two countries’ sets of AWSs. The temperature drops observed in the UK and Iceland are comparable with the results of the previous analyses referenced above (although our study uses far more stations and has a much more extensive spatial coverage than most of these). Despite the Faroes being directly in the path of the total eclipse zone, the islands’ AWS data show much more limited evidence of an eclipse-related meteorological signature, which we attribute to a combination of generally cloudy conditions there and the extreme maritime location of its weather stations, with little or no continental heating/cooling as may be the case in the UK and Iceland.

## Supplementary Material

Supplement Tables and Figures
