# Supplementary material for: Meteorological effects of the solar eclipse of 20 March 2015: analysis of UK Met Office automatic weather station data and comparison with automatic weather station data from the Faroes and Iceland
Source: Philos Trans A Math Phys Eng Sci. 2016 Sep 28;374(2077):20150212. doi: 10.1098/rsta.2015.0212 (PMC5004046; doi:10.1098/rsta.2015.0212)

Table S1: One-minute ~~T~~temperature (°C) at 15-min intervals (UTC) during the eclipse period on 20 March 2015.

Time → Station ↓	0830	0845	0900	0915	0930	0945	1000	1015	1030
3/Fair Isle	6.2	6.6	7.0	6.8	6.5	6.4	6.6	6.8	7.0
9/Lerwick	6.2	6.6	6.7	6.1	6.5	6.5	6.4	5.6	6.4
12/Baltasound	7.0	6.9	7.0	7.2	6.8	6.4	6.5	6.8	7.5
23/Kirkwall	6.8	7.2	7.5	7.2	7.0	6.9	6.9	7.3	7.6
32/Wick	7.1	6.7	6.8	7.1	6.8	6.2	6.5	6.8	7.7
44/Altnaharra	6.4	6.8	7.3	7.3	6.5	6.4	6.5	6.8	7.0
54/Stornoway	7.4	7.8	8.3	7.5	7.3	7.2	7.2	7.7	7.0
67/Loch Glascarnoch	5.4	5.8	5.5	5.4	5.0	5.0	4.8	5.1	5.6
113/Aviemore	8.4	8.6	8.4	8.1	7.5	6.9	6.8	7.3	7.9
132/Kinloss	7.7	7.9	8.1	7.9	7.7	7.1	7.0	7.2	8.0
137/Lossiemouth	7.9	7.7	7.6	8.0	7.7	7.1	7.1	7.3	7.8
150/Aboyne	10.4	11.0	10.5	10.5	10.1	9.5	9.9	10.3	8.5
161/Dyce	10.2	10.3	10.2	10.4	10.2	10.0	8.1	8.2	8.5
177/Inverbervie	7.4	8.2	8.2	8.4	8.5	7.9	7.9	9.1	8.2
235/Leuchars	7.9	9.4	9.7	9.3	8.7	8.5	9.0	10.0	11.1
268/Charterhall	8.3	8.9	9.0	8.9	8.2	7.4	8.2	8.9	9.4
315/Boulmer	9.7	10.5	10.6	9.8	9.7	9.2	9.6	10.0	10.5
326/Durham	8.9	9.5	9.7	9.6	9.2	8.8	8.9	9.5	10.9
346/Linton on Ouse	7.7	8.2	8.9	8.7	8.3	8.3	8.2	8.5	9.3
360/Scarborough	7.3	7.6	7.4	6.6	5.7	5.4	5.7	6.5	7.8
384/Waddington	4.6	4.8	5.4	5.4	5.0	4.9	5.6	6.6	7.5
386/Cranwell	4.7	5.3	5.6	5.6	5.0	4.5	5.4	6.4	7.5
393/Coningsby	4.7	4.8	4.8	5.2	4.8	4.5	5.4	6.0	6.8
409/Marham	4.3	4.5	4.5	4.5	4.4	4.4	4.6	4.8	4.9
421/Weybourne	5.0	5.2	5.2	5.4	5.1	4.9	5.3	5.7	6.1
426/Cromer	4.5	4.7	4.4	4.4	4.3	4.5	5.0	5.5	6.0
471/Rothamsted	3.7	3.7	3.7	3.7	3.6	3.6	3.9	4.2	4.6
525/Sheffield	9.0	9.0	8.9	9.3	9.5	9.5	9.6	9.9	10.3
578/Northampton, Moulton	4.9	5.0	4.7	4.7	4.5	4.3	5.0	5.6	6.7
605/Brize Norton	5.6	5.9	5.9	5.5	5.4	5.4	5.8	6.7	6.7
613/Benson	4.4	4.5	4.5	4.4	4.3	4.3	4.5	4.9	5.4
622/Keele	4.5	5.4	5.8	5.9	5.6	5.1	5.5	6.8	8.1
643/Shawbury	5.5	5.9	5.8	5.5	4.7	4.5	5.4	6.5	7.3
657/Pershore	4.6	5.2	5.5	5.6	5.5	5.5	6.0	6.6	7.4
676/Filton	4.1	4.3	4.4	4.4	4.3	4.5	5.1	6.2	6.7
697/London, St. James's Park	5.3	5.4	5.3	5.4	5.3	5.2	5.4	5.6	5.7
708/Heathrow	4.9	4.8	5.0	4.7	4.7	4.8	4.9	5.0	5.1
709/Northolt	4.5	4.5	4.6	4.5	4.5	4.4	4.6	4.7	4.8
723/Kew Gardens	5.1	5.1	5.0	4.9	4.8	4.8	4.9	4.8	5.1
775/Manston	4.9	4.8	4.9	4.7	4.4	4.5	4.6	4.6	4.7
795/Shoreham	5.1	5.0	5.0	4.8	4.7	4.8	4.9	5.1	5.3

811/Herstmonceux	4.8	4.9	4.8	4.7	4.6	4.6	4.8	5.3	5.3
830/Reading	4.5	4.4	4.4	4.4	4.3	4.4	4.4	4.4	4.5
842/Hurn	5.1	4.9	5.0	5.0	4.7	4.8	5.0	5.1	5.6
862/Odiham	4.0	3.9	4.0	3.9	3.8	3.8	3.9	3.9	4.8
876/Isle of Wight, St Catherine's Point	5.5	5.4	5.5	5.4	5.3	5.3	5.4	5.7	5.9
888/Larkhill	4.8	4.7	4.7	4.6	4.4	4.1	4.7	5.6	6.0
889/Boscombe	4.6	4.6	4.4	4.3	4.3	4.4	4.5	4.6	5.5
1023/Eskdalemuir	6.9	7.0	7.0	7.0	6.9	6.7	6.9	7.8	7.9
1046/Ronaldsway	9.2	9.1	8.9	8.5	8.4	8.5	8.5	8.8	9.2
1060/Keswick	7.5	7.3	8.1	8.0	7.8	7.7	8.1	8.4	9.2
1083/Shap	8.6	8.9	8.7	8.8	8.2	7.8	7.6	7.5	8.1
1145/Valley	8.1	7.7	7.8	7.5	7.3	7.4	7.5	7.6	7.9
1198/Aberporth	5.8	6.5	6.3	5.9	4.9	3.1	3.5	6.1	7.7
1226/Pembrey Sands	4.6	5.2	5.4	5.4	4.6	4.3	5.5	6.4	7.2
1255/Mumbles	6.5	6.2	6.3	5.7	5.4	5.8	6.7	8.1	8.9
1302/Yeovilton	6.1	6.2	6.2	6.1	6.1	6.2	6.2	6.5	6.6
1319/Isle of Portland	5.2	5.2	5.1	5.0	4.9	5.1	5.2	5.5	5.8
1326/Swanage	4.9	4.9	5.0	5.1	5.1	5.2	5.2	5.2	5.1
1336/Plymouth Mountbatten	4.9	5.3	5.2	4.8	4.1	4.1	5.2	5.5	6.2
1346/Chivenor	3.1	3.3	3.4	3.6	3.2	3.4	4.2	5.2	6.4
1386/Scilly	8.2	8.2	8.2	8.0	7.8	8.0	8.4	8.7	9.3
1395/Camborne	5.8	6.1	6.5	6.4	6.1	6.3	6.9	7.5	7.9
1450/Aldergrove	7.6	7.7	7.9	7.8	7.3	7.4	7.7	7.6	8.0
16588/Gravesend	4.8	4.9	4.9	5.0	4.8	4.7	4.7	4.8	5.0
17309/Crosby	7.2	7.5	7.7	7.0	7.1	7.0	7.0	7.1	7.3
17314/Leeming	5.9	6.2	6.5	6.6	6.6	6.6	6.9	7.7	8.5
18903/South Uist Range	7.5	7.4	7.6	7.4	7.0	7.1	6.9	7.6	8.2
18974/Tiree	8.0	8.4	8.3	8.1	7.9	7.8	7.3	8.0	8.6
19187/Coleshill	5.3	5.7	6.3	6.2	6.0	5.8	6.1	7.3	8.3
19260/Edinburgh	9.2	9.4	9.3	9.3	9.0	8.8	9.0	9.7	9.6
25727/Southampton	5.2	5.2	5.2	5.3	5.3	5.3	5.5	5.4	5.3
30620/Charlwood	4.4	4.4	4.4	4.4	4.3	4.3	4.5	4.8	5.1
55827/Braemar	8.3	8.1	8.0	7.9	7.9	7.6	7.6	7.9	8.7
56937/Giant's Causeway	7.3	7.2	7.5	7.4	7.3	7.3	7.7	7.8	7.7
56958/Emley Moor	6.9	7.1	7.0	6.7	6.1	6.2	7.0	7.2	7.3
MEAN	6.22	6.44	6.51	6.41	6.16	6.02	6.27	6.72	7.19

Table S2: One-minute ~~W~~wind speed (knots) at 15-min intervals (UTC) during the eclipse period on 20 March 2015. NA = data Not Available.

Time → Station ↓	0830	0845	0900	0915	0930	0945	1000	1015	1030
3/Fair Isle	21.0	14.7	15.9	18.4	18.8	11.1	17.9	14.2	13.4
9/Lerwick	15.0	19.8	19.3	18.7	16.4	22.3	17.8	20.3	19.8
12/Baltasound	17.0	16.6	17.8	18.9	23.3	19.9	19.8	15.2	22.1
23/Kirkwall	15.3	16.2	17.5	22.4	18.3	17.4	18.3	15.4	16.6
32/Wick	14.0	19.9	21.1	18.8	18.0	17.2	13.3	15.1	20.6
44/Altnaharra	18.7	16.3	18.0	17.2	13.5	7.6	8.3	13.0	16.8
54/Stornoway	9.6	9.8	11.6	13.8	15.7	11.2	12.6	8.6	12.3
67/Loch Glascarnoch	17.4	15.1	14.4	12.7	12.1	14.2	11.6	10.7	11.4
113/Aviemore	2.5	2.9	1.8	4.5	4.0	1.5	1.3	4.5	3.6
132/Kinloss	10.1	6.8	12.3	11.6	6.4	9.6	12.7	9.2	14.1
137/Lossiemouth	12.3	9.6	16.6	16.9	11.8	16.9	15.3	16.1	13.5
150/Aboyne	9.6	9.9	14.0	13.2	13.7	9.0	7.9	11.9	14.9
161/Dyce	6.4	6.0	9.4	13.7	9.6	10.8	11.1	9.9	6.5
177/Inverbervie	NA	NA	NA	NA	NA	NA	NA	NA	NA
235/Leuchars	13.0	15.3	14.3	11.9	12.9	11.5	9.8	11.5	11.5
268/Charterhall	7.4	12.7	11.9	8.8	7.0	6.6	5.3	6.2	7.9
315/Boulmer	2.8	4.4	10.5	8.4	5.8	7.5	7.4	8.6	5.3
326/Durham	NA	NA	NA	NA	NA	NA	NA	NA	NA
346/Linton on Ouse	5.8	6.2	6.3	7.2	6.2	4.9	4.5	4.3	4.9
360/Scarborough	NA	NA	NA	NA	NA	NA	NA	NA	NA
384/Waddington	6.2	6.1	6.4	5.1	5.0	4.5	5.4	6.5	7.8
386/Cranwell	8.4	9.7	9.6	9.1	9.5	8.1	5.9	7.3	8.2
393/Coningsby	7.1	6.5	5.9	4.7	6.0	5.9	4.9	7.7	6.8
409/Marham	1.8	3.1	4.8	2.7	3.5	4.3	3.4	5.4	4.9
421/Weybourne	5.5	3.6	5.4	6.1	5.8	6.9	5.3	6.5	6.2
426/Cromer	NA	NA	NA	NA	NA	NA	NA	NA	NA
471/Rothamsted	NA	NA	NA	NA	NA	NA	NA	NA	NA
525/Sheffield	NA	NA	NA	NA	NA	NA	NA	NA	NA
578/Northampton, Moulton	NA	NA	NA	NA	NA	NA	NA	NA	NA
605/Brize Norton	4.8	4.0	4.0	2.9	2.4	2.9	1.8	2.4	0.5
613/Benson	5.9	3.0	4.3	5.2	6.3	4.7	3.5	3.3	4.0
622/Keele	NA	NA	NA	NA	NA	NA	NA	NA	NA
643/Shawbury	4.6	5.6	3.4	3.1	2.7	3.6	2.8	5.7	5.2
657/Pershore	2.3	1.5	1.6	2.5	3.6	2.2	2.9	3.4	2.8
676/Filton	1.7	1.9	2.3	2.2	2.6	0.7	3.0	3.4	5.6
697/London, St. James's Park	NA	NA	NA	NA	NA	NA	NA	NA	NA
708/Heathrow	5.0	4.4	4.9	4.0	3.8	4.3	3.0	3.8	2.7
709/Northolt	6.7	6.3	6.3	2.9	4.2	3.5	4.2	5.3	3.8
723/Kew Gardens	5.0	3.3	3.5	3.2	1.6	1.6	4.4	2.9	3.1
775/Manston	8.3	9.0	8.5	10.9	8.2	9.5	8.7	9.1	7.4
795/Shoreham	6.0	9.0	5.9	7.3	7.7	6.5	8.0	8.5	8.1

811/Herstmonceux	5.2	5.8	4.7	5.4	4.4	4.0	7.2	7.0	8.2
830/Reading	NA	NA	NA	NA	NA	NA	NA	NA	NA
842/Hurn	7.9	8.3	8.8	7.4	6.6	5.7	7.0	7.8	7.4
862/Odiham	8.7	6.8	7.1	7.2	6.8	7.1	5.4	6.3	4.8
876/Isle of Wight, St Catherine’s Point	6.7	6.7	4.3	3.9	6.6	1.6	4.0	3.7	9.1
888/Larkhill	7.9	8.7	8.7	7.6	8.0	6.3	7.7	7.5	7.0
889/Boscombe	7.2	5.5	8.7	8.0	7.5	7.2	5.0	5.7	6.6
1023/Eskdalemuir	8.9	8.9	4.4	5.7	7.4	8.2	6.9	6.2	8.8
1046/Ronaldsway	10.1	9.7	8.8	7.1	7.0	10.4	9.5	8.9	9.6
1060/Keswick	2.2	2.3	8.4	4.6	4.8	4.2	5.4	5.3	6.6
1083/Shap	4.7	4.6	4.5	6.6	6.6	5.8	6.4	6.2	6.4
1145/Valley	3.7	5.2	4.1	4.9	5.2	5.7	4.0	3.7	3.9
1198/Aberporth	1.1	0.1	1.6	1.7	1.3	1.4	1.7	1.3	0.9
1226/Pembrey Sands	2.0	2.5	2.5	2.3	2.1	4.4	4.1	5.2	4.6
1255/Mumbles	5.8	5.4	4.8	4.6	3.0	3.1	2.1	2.1	1.4
1302/Yeovilton	7.2	5.4	5.8	6.0	4.3	4.3	5.4	4.7	3.5
1319/Isle of Portland	8.9	7.0	7.2	9.8	8.2	9.0	9.9	10.3	9.4
1326/Swanage	NA	NA	NA	NA	NA	NA	NA	NA	NA
1336/Plymouth Mountbatten	1.7	0.8	1.7	5.4	3.0	3.2	3.0	4.0	2.2
1346/Chivenor	2.5	3.6	2.8	2.2	3.9	2.7	1.8	1.8	1.8
1386/Scilly	12.0	8.1	9.4	9.4	11.8	11.4	12.2	12.6	12.1
1395/Camborne	4.6	4.0	6.6	5.9	8.2	7.9	7.0	8.6	10.5
1450/Aldergrove	5.1	7.8	7.3	6.6	6.6	7.2	5.0	7.9	7.2
16588/Gravesend	4.4	5.0	4.1	4.6	4.2	4.6	3.7	4.5	4.2
17309/Crosby	5.6	5.6	7.3	7.3	9.5	12.5	10.8	7.9	8.6
17314/Leeming	3.8	3.9	5.9	5.9	5.8	2.9	2.1	0.9	2.4
18903/South Uist Range	13.5	12.4	9.7	12.2	10.0	12.0	10.4	11.8	12.5
18974/Tiree	10.4	6.8	8.4	8.6	9.4	12.0	10.5	11.5	12.2
19187/Coleshill	5.1	2.3	1.4	2.0	0.1	0.5	0.6	2.8	2.8
19260/Edinburgh	11.0	13.0	8.7	11.0	8.1	11.0	10.0	12.0	11.0
25727/Southampton	3.5	5.8	5.4	4.6	3.2	3.7	2.8	5.6	5.3
30620/Charlwood	6.5	7.8	4.9	6.0	4.2	4.9	5.0	5.6	6.0
55827/Braemar	NA	NA	NA	NA	NA	NA	NA	NA	NA
56937/Giant’s Causeway	NA	NA	NA	NA	NA	NA	NA	NA	NA
56958/Emley Moor	7.7	11.2	13.1	12.7	11.5	13.8	13.5	12.9	13.9
MEAN	7.59	7.46	7.95	8.03	7.55	7.41	7.18	7.59	7.99

Table S3: One-minute ~~W~~wind direction (degrees) at 15-min intervals (UTC) during the eclipse period on 20 March 2015. NA = data Not Available.

Time → Station ↓	0830	0845	0900	0915	0930	0945	1000	1015	1030
3/Fair Isle	283.4	283.2	282.3	273.0	282.1	269.9	263.0	268.0	277.8
9/Lerwick	283.7	276.9	275.6	287.0	287.3	286.5	285.6	289.3	288.6
12/Baltasound	285.8	274.8	271.7	282.1	289.5	286.0	292.3	294.6	295.1
23/Kirkwall	287.7	287.9	285.7	285.6	278.7	279.3	281.2	280.0	283.5
32/Wick	266.9	282.0	283.9	279.3	283.1	274.0	269.5	277.5	290.8
44/Altnaharra	268.2	262.2	247.8	250.8	249.0	252.6	239.6	254.8	258.4
54/Stornoway	256.1	258.5	265.5	271.6	280.5	273.5	269.8	290.3	288.7
67/Loch Glascarnoch	289.9	300.6	280.4	267.2	287.4	292.2	286.1	289.0	297.8
113/Aviemore	190.3	198.9	166.4	245.6	336.5	22.6	66.9	286.8	250.1
132/Kinloss	298.4	264.8	287.2	271.5	265.5	302.1	300.7	284.4	281.7
137/Lossiemouth	295.1	292.6	299.6	289.6	285.7	296.9	291.4	292.7	295.6
150/Aboyne	265.1	285.4	292.8	295.7	300.3	286.4	284.5	289.4	317.0
161/Dyce	295.8	316.3	309.2	306.9	321.1	309.8	329.4	330.0	327.8
177/Inverbervie	NA	NA	NA	NA	NA	NA	NA	NA	NA
235/Leuchars	265.3	262.6	249.7	261.3	255.8	257.8	260.6	258.4	256.3
268/Charterhall	243.9	233.2	238.1	253.1	237.7	189.3	208.3	190.6	229.0
315/Boulmer	260.2	287.3	273.2	254.3	262.3	256.0	263.5	272.8	224.8
326/Durham	NA	NA	NA	NA	NA	NA	NA	NA	NA
346/Linton on Ouse	236.2	206.6	234.8	249.5	244.5	220.3	213.7	210.4	208.0
360/Scarborough	NA	NA	NA	NA	NA	NA	NA	NA	NA
384/Waddington	245.4	250.3	243.8	240.4	218.7	227.8	217.1	226.6	233.6
386/Cranwell	247.3	253.4	260.9	258.9	242.9	235.5	223.8	231.4	236.5
393/Coningsby	202.6	221.0	212.7	226.5	206.0	194.8	208.3	212.0	199.4
409/Marham	285.4	276.4	290.1	291.1	235.5	218.3	217.1	210.3	230.9
421/Weybourne	268.4	287.6	284.3	257.8	255.8	232.0	241.6	229.1	237.2
426/Cromer	NA	NA	NA	NA	NA	NA	NA	NA	NA
471/Rothamsted	NA	NA	NA	NA	NA	NA	NA	NA	NA
525/Sheffield	NA	NA	NA	NA	NA	NA	NA	NA	NA
578/Northampton, Moulton	NA	NA	NA	NA	NA	NA	NA	NA	NA
605/Brize Norton	50.9	32.2	62.1	75.0	58.3	55.3	44.8	71.6	65.7
613/Benson	356.3	15.5	5.7	2.3	355.0	21.7	350.2	55.4	319.8
622/Keele	NA	NA	NA	NA	NA	NA	NA	NA	NA
643/Shawbury	256.7	241.9	234.7	216.3	228.9	213.7	222.3	249.6	238.2
657/Pershore	262.1	290.5	323.5	312.4	315.9	274.6	297.3	287.7	323.4
676/Filton	7.9	2.8	16.2	22.5	33.1	64.4	21.3	78.8	17.4
697/London, St. James's Park	NA	NA	NA	NA	NA	NA	NA	NA	NA
708/Heathrow	12.7	79.7	20.7	44.5	37.4	20.9	355.9	345.8	10.2
709/Northolt	17.2	48.5	71.9	86.1	9.7	359.6	17.4	50.7	24.6
723/Kew Gardens	330.8	359.2	12.0	341.2	342.8	356.3	13.9	344.2	56.3
775/Manston	14.3	12.1	7.5	26.5	356.1	14.6	43.0	8.8	1.7
795/Shoreham	349.4	2.7	12.3	17.8	2.7	10.2	17.5	16.4	9.0

811/Herstmonceux	17.8	15.7	22.6	14.6	39.0	43.5	53.3	15.1	15.1
830/Reading	NA	NA	NA	NA	NA	NA	NA	NA	NA
842/Hurn	47.4	49.9	15.9	11.1	22.9	27.6	23.1	5.0	40.8
862/Odiham	38.0	36.2	34.4	20.0	53.0	38.8	37.1	8.0	40.3
876/Isle of Wight, St Catherine's Point	54.7	54.6	52.1	57.2	66.3	72.3	81.8	72.0	72.2
888/Larkhill	46.4	52.6	60.3	58.5	48.2	49.0	45.5	49.6	50.3
889/Boscombe	35.7	50.4	13.6	14.9	13.1	64.3	30.4	16.5	16.3
1023/Eskdalemuir	230.7	224.8	219.1	187.2	193.9	202.0	239.5	234.4	225.3
1046/Ronaldsway	289.7	293.6	290.6	291.1	297.4	288.0	282.5	283.6	288.8
1060/Keswick	155	231.4	266.4	273.9	279.9	287.0	279.9	284.2	266.6
1083/Shap	245.0	301.8	311.5	286.7	293.0	304.5	335.1	318.8	321.8
1145/Valley	233.6	189.8	214.2	193.8	201.8	200.9	196.4	224.8	237.1
1198/Aberporth	329.1	341.7	315.5	268.9	260.9	247.4	218.3	214.7	27.8
1226/Pembrey Sands	59.7	66.5	44.5	36.5	353.2	343.7	346.1	20.2	335.0
1255/Mumbles	22.8	359.4	25.8	7.1	17.7	24.7	329.8	173.0	220.9
1302/Yeovilton	26.9	38.0	29.9	31.6	23.7	340.6	320.8	332.5	359.0
1319/Isle of Portland	54.9	24.1	20.5	19.5	23.0	51.3	58.3	41.0	61.5
1326/Swanage	NA	NA	NA	NA	NA	NA	NA	NA	NA
1336/Plymouth Mountbatten	311.2	4.4	308.7	254.7	275.4	275.5	275.3	320.1	306.8
1346/Chivenor	114.9	84.9	97.7	98.7	93.8	72.7	14.8	158.6	223.3
1386/Scilly	13.9	17.9	15.1	14.6	19.6	21.7	14.5	7.5	22.6
1395/Camborne	59.3	54.3	46.6	38.6	23.0	26.6	42.4	25.6	28.9
1450/Aldergrove	288.1	303.3	297.6	301.5	307.5	302.4	287.1	294.8	295.4
16588/Gravesend	35.6	17.0	69.9	43.6	2.3	24.7	9.4	9.6	63.0
17309/Crosby	239.9	247.5	247.9	239.6	260.5	270.7	272.9	267.6	257.7
17314/Leeming	170.2	169.4	156.5	163.3	171.3	182.7	197.2	195.6	127.9
18903/South Uist Range	292.1	277.3	273.5	292.0	296.9	296.7	316.4	304.8	299.8
18974/Tiree	318.8	322.9	306.3	306.2	305.1	302.8	293.3	300.3	313.9
19187/Coleshill	312.9	306.1	320.0	294.5	345.3	288.6	8.6	279.7	273.8
19260/Edinburgh	248.9	233.5	254.5	249.2	252.6	258.8	253.0	242.8	244.1
25727/Southampton	32.4	17.6	42.6	60.7	45.0	51.3	45.2	51.4	8.5
30620/Charlwood	48.5	61.2	45.9	30.2	30.8	23.8	44.4	20.1	42.5
55827/Braemar	NA	NA	NA	NA	NA	NA	NA	NA	NA
56937/Giant's Causeway	NA	NA	NA	NA	NA	NA	NA	NA	NA
56958/Emley Moor	266.6	267.4	271.8	276.8	276.3	269.5	273.9	282.4	281.4

Table S4: One-minute ~~M~~mean sea-level pressure (tenths of mb, last 3 digits with decimal point omitted) at 15-min intervals (UTC) during the eclipse on 20 March 2015. NA = data Not Available.

Time → Station ↓	0830	0845	0900	0915	0930	0945	1000	1015	1030
3/Fair Isle	194	193	191	192	191	189	188	186	185
9/Lerwick	176	175	173	172	171	168	167	167	165
12/Baltasound	165	159	159	157	157	158	157	155	153
23/Kirkwall	216	215	212	213	211	211	212	210	208
32/Wick	226	225	223	224	223	222	221	219	217
44/Altnaharra	240	239	240	240	240	239	238	236	236
54/Stornoway	254	253	254	255	253	252	252	252	252
67/Loch Glascarnoch	258	256	257	255	254	258	257	254	252
113/Aviemore	248	247	246	247	248	249	249	249	248
132/Kinloss	245	244	245	245	246	246	245	243	241
137/Lossiemouth	243	243	242	243	243	243	242	240	240
150/Aboyne	237	235	235	234	233	232	232	232	235
161/Dyce	237	237	235	233	233	232	235	234	232
177/Inverbervie	242	239	240	238	235	236	234	232	233
235/Leuchars	247	247	245	244	242	242	240	240	238
268/Charterhall	255	253	254	254	254	254	251	248	246
315/Boulmer	257	255	256	257	257	256	255	253	251
326/Durham	NA	NA	NA	NA	NA	NA	NA	NA	NA
346/Linton on Ouse	280	279	277	275	274	273	273	272	271
360/Scarborough	NA	NA	NA	NA	NA	NA	NA	NA	NA
384/Waddington	292	290	289	287	285	285	284	281	279
386/Cranwell	292	291	291	289	288	287	286	284	281
393/Coningsby	290	289	289	289	287	284	285	284	280
409/Marham	295	296	294	292	292	290	288	287	286
421/Weybourne	293	292	290	288	288	285	282	281	280
426/Cromer	NA	NA	NA	NA	NA	NA	NA	NA	NA
471/Rothamsted	301	301	300	298	299	297	296	293	292
525/Sheffield	NA	NA	NA	NA	NA	NA	NA	NA	NA
578/Northampton, Moulton	NA	NA	NA	NA	NA	NA	NA	NA	NA
605/Brize Norton	300	299	298	298	298	297	296	293	291
613/Benson	302	301	300	298	298	297	296	295	294
622/Keele	NA	NA	NA	NA	NA	NA	NA	NA	NA
643/Shawbury	300	299	297	296	296	295	293	291	290
657/Pershore	307	307	306	304	303	301	300	299	297
676/Filton	302	301	302	301	301	298	298	296	295
697/London, St. James's Park	NA	NA	NA	NA	NA	NA	NA	NA	NA
708/Heathrow	299	299	298	297	295	295	293	292	292
709/Northolt	299	299	298	297	295	294	293	293	292
723/Kew Gardens	NA	NA	NA	NA	NA	NA	NA	NA	NA
775/Manston	290	290	289	289	289	289	287	288	286

795/Shoreham	292	291	291	290	290	290	289	288	286
811/Herstmonceux	288	289	289	289	287	286	285	285	284
830/Reading	NA	NA	NA	NA	NA	NA	NA	NA	NA
842/Hurn	297	298	296	296	296	294	292	291	290
862/Odiham	298	298	297	296	295	294	293	293	290
876/Isle of Wight, St Catherine’s Point	290	289	290	290	290	289	287	286	284
888/Larkhill	299	299	298	297	297	295	295	294	293
889/Boscombe	300	300	299	297	297	295	296	294	293
1023/Eskdalemuir	273	274	273	273	273	273	269	267	264
1046/Ronaldsway	291	292	291	292	291	291	289	288	286
1060/Keswick	NA	285	284	284	282	281	280	280	278
1083/Shap	279	279	279	279	280	280	279	279	276
1145/Valley	301	298	299	300	299	300	300	300	299
1198/Aberporth	306	305	305	305	304	305	304	303	300
1226/Pembrey Sands	308	307	306	304	303	302	302	300	300
1255/Mumbles	310	310	309	308	307	306	305	303	302
13023/Yeovilton	300	299	299	298	298	297	296	295	293
1319/Isle of Portland	297	298	297	296	295	293	292	291	291
1326/Swanage	NA	NA	NA	NA	NA	NA	NA	NA	NA
1336/Plymouth Mountbatten	303	302	301	300	300	298	297	295	294
1346/Chivenor	306	306	304	304	304	303	302	300	298
1386/Scilly	306	304	304	303	303	302	303	302	302
1395/Camborne	303	303	302	302	300	298	298	297	295
1450/Aldergrove	299	297	298	297	296	296	295	294	294
16588/Gravesend	294	294	293	292	291	289	288	287	286
17309/Crosby	295	294	292	292	291	290	291	291	291
17314/Leeming	279	277	276	274	272	272	272	271	270
18903/South Uist Range	273	273	272	273	272	271	270	269	268
18974/Tiree	282	280	279	280	282	283	281	280	280
19187/Coleshill	298	297	295	294	293	291	291	289	287
19260/Edinburgh	259	259	259	257	254	255	253	251	250
25727/Southampton	NA	NA	NA	NA	NA	NA	NA	NA	NA
30620/Charlwood	294	293	292	292	292	290	289	287	286
55827/Braemar	NA	NA	NA	NA	NA	NA	NA	NA	NA
56937/Giant’s Causeway	NA	NA	NA	NA	NA	NA	NA	NA	NA
56958/Emley Moor	NA	NA	NA	NA	NA	NA	NA	NA	NA
MEAN (mb+1000)	27.71	27.64	27.57	27.51	27.44	27.36	27.27	27.14	27.01

Table S5: One-minute ~~C~~cloud cover (oktas) and height (feet) of up to 3 different levels of cloud (if present) at 15-min intervals (UTC) during the eclipse on 20 March 2015.

Time →<br>Station ↓	0830	0845	0900	0915	0930	0945	1000	1015	1030
9/Lerwick	8<br>3020	8<br>2390<br>5450	8<br>1280<br>2590	8<br>4130	7<br>5350	8<br>1670	8<br>1150<br>2070	8<br>3540	8<br>2170<br>5910<br>6960
23/Kirkwall	6	3<br>1850	5<br>1150	1	0	5<br>2150	6<br>4600	7<br>1750	7<br>5950
32/Wick	8<br>1550	8<br>4050	6<br>4700	7<br>2200	7<br>4900	6	5	2	2<br>3950
54/Stornoway	7<br>4650	7<br>2100	7<br>1380	7<br>2310	7<br>3000	8<br>1050<br>3300	8<br>1140<br>4260	8<br>960	8<br>4860
67/Loch Glascarnoch	8<br>1180	8<br>1280<br>2200<br>3280	8<br>1340<br>2230	8<br>1800<br>2720	8<br>2030<br>2820<br>3540	8<br>1800	8<br>1020	8<br>1080<br>2850<br>4130	8<br>3120
113/Aviemore	8<br>2690	8<br>2030	8<br>2590	8<br>2690	8<br>1570	8<br>2130	8<br>1480	8<br>2490<br>4070	8<br>2100<br>4490
132/Kinloss	8<br>3180	8<br>3280	8<br>2850	7<br>3280	7<br>2850	8<br>2170	8<br>2890	8<br>4100<br>4760	7<br>4890<br>5540
137/Lossiemouth	8<br>3180	7<br>3310	8<br>3180	7<br>3440	7<br>2890<br>3540	8<br>3120	8<br>3150	7<br>3810	8<br>5180
150/Aboyne	3	5<br>2890	6<br>2690	5<br>3900	6<br>2590<br>14170	7<br>15020	6	6<br>4920	7<br>1900
161/Dyce	8<br>6480	8<br>2100	6	5<br>2010	7<br>2040	7<br>2430	7<br>1680	8<br>1170	8<br>1890<br>2880
177/Inverbervie	8<br>6820	8<br>6860	8<br>6760	8<br>6920	8<br>6820	8<br>6820	7	7	5<br>3410
235/Leuchars	1	2	1	1	1	0	0	0	0
268/Charterhall	6<br>2200	3	2	2	1	0	1	0	1
315/Boulmer	7<br>5650	7<br>7200	5	5	6<br>6350	6	7<br>6750	3	2
346/Linton on Ouse	7<br>6450	7<br>6270	8<br>5700	8<br>5340	8<br>4890	8<br>4830	8<br>5880	8<br>5880	8<br>4350<br>5880
384/Waddington	0	0	0	0	0	0	0	0	0
386/Cranwell	3	1	1	1	0	0	0	0	0

393/Coningsby	6<br>8220<br>9060	6<br>8190	3	1	1	0	1<br>9600	1	1
409/Marham	8<br>750	8<br>810	8<br>810	8<br>930	8<br>900	8<br>930	8<br>930	8<br>930	8<br>840
421/Weybourne	8<br>950	8<br>980	8<br>950	8<br>920	8<br>920	8<br>920	8<br>820<br>11190	8<br>950	8<br>950
605/Brize Norton	8<br>1100	7<br>1200	7<br>8500	8<br>1200	8<br>1150	8<br>1200	8<br>7900	8<br>7800	7<br>11750
613/Benson	8<br>1020	8<br>1080	8<br>1080	8<br>1080	8<br>1080	8<br>1250	8<br>1050	8<br>1250	8<br>1350
643/Shawbury	8<br>5550	8<br>5400	8<br>5500	4	2	1	1	0	0
657/Pershore	7<br>8400	7<br>8500	8<br>8140	8<br>7870	8<br>8400	8<br>8270	8<br>8100	8<br>8140	8<br>8140
676/Filton	1	1	1	1	0	0	0	0	0
708/Heathrow	8<br>750	8<br>900	8<br>750	8<br>800	8<br>800	8<br>750	8<br>800	8<br>700	8<br>900
709/Northolt	8<br>650	8<br>750	8<br>750	8<br>800	8<br>650	8<br>700	8<br>800	8<br>800	8<br>700
775/Manston	8<br>1100	8<br>900	8<br>850	8<br>850	8<br>850	8<br>950	8<br>900	8<br>800	8<br>850
811/Herstmonceux	8<br>1150	8<br>1150	8<br>1300	8<br>1200	8<br>1200	8<br>1300	8<br>1300	8<br>1100	8<br>1200
842/Hurn	8<br>1150	8<br>1150	8<br>1100	8<br>1100	8<br>1100	8<br>1050	8<br>1050	8<br>1050	8<br>1050
862/Odiham	8<br>620	8<br>590	8<br>560	8<br>560	8<br>520	8<br>520	8<br>520	8<br>560	8<br>560
888/Larkhill	8<br>1080	8<br>1140	8<br>1170	8<br>1140	8<br>1140	7	3	3<br>1110	4<br>1170
889/Boscombe	8<br>950	8<br>950	8<br>1020	8<br>920	8<br>950	8<br>950	8<br>1020	8<br>950	8<br>1150
1023/Eskdalemuir	8<br>1980	8<br>1470	8<br>1470	8<br>1410	8<br>1320	7<br>1410	8<br>1350	8<br>1380	8<br>1080
1046/Ronaldsway	8<br>2850	8<br>2850	8<br>1650<br>3300	8<br>600<br>1860	8<br>930	8<br>1020<br>4020	8<br>930<br>3960<br>4620	8<br>990<br>3990	8<br>3900
1060/Keswick	7<br>1140	7<br>1050	7<br>960	8<br>1050<br>3150	8<br>990<br>3240	8<br>3690<br>5190	8<br>1050<br>3600	8<br>1020	8<br>1110
1083/Shap	8<br>2030	8<br>2890<br>5540	8<br>1870<br>5380	8<br>2990<br>3740	8<br>1900<br>3380	8<br>1440	7<br>1710	7<br>1900<br>2560	7<br>1840
1145/Valley	7<br>5940	7<br>1800<br>6000	8<br>1650	8<br>930	8<br>750	8<br>660	7<br>660	7<br>660	8<br>570

1198/Aberporth	0	0	0	0	0	0	0	0	3<br>5250
1226/Pembrey Sands	0	0	0	0	0	0	0	0	0
1302/Yeovilton	8<br>1480	8<br>1440	8<br>1310	8<br>1280	8<br>1410	8<br>1340	8<br>1340	8<br>1340	8<br>1310
1319/Isle of Portland	8<br>1210	8<br>1210	8<br>1180	8<br>1210	8<br>1210	8<br>1380	8<br>1280	8<br>1210	8<br>1250
1336/Plymouth Mountbatten	1	1	1	1	2	1	3<br>11580	2	1
1346/Chivenor	0	0	0	0	0	0	0	0	0
1386/Scilly	8<br>1280	8<br>1210	7	7<br>950	8<br>790	5	4	3	5<br>1080
1395/Camborne	6	3	4	5	3	1	1	0	1
1450/Aldergrove	7<br>3210	7<br>3510	7<br>2530	8<br>2690	8<br>2620	8<br>3210	7<br>2760	8<br>2360	8<br>3120
17309/Crosby	7<br>5850	7<br>5940	7<br>1380	8<br>1530<br>6030	8<br>3510	8<br>2490	8<br>2460	8<br>2610	7<br>2910
17314/Leeming	8<br>5850	8<br>6000	8<br>6180	8<br>6270	8<br>6330	8<br>6510	8<br>6630	8<br>6630	8<br>3030<br>6660
18903/South Uist Range	8<br>1770	8<br>4740	8<br>1710<br>2400<br>3900	8<br>1800	8<br>4020	7	8<br>3270	7<br>3810	4<br>3630
18974/Tiree	8<br>3600	7<br>3800	8<br>3600	7<br>3250	8<br>3500	7<br>2450	8<br>3150	8<br>4100	6<br>2550
19187/Coleshill	7<br>7710	4<br>8070	6<br>7910	6<br>8140	5	2	1	1	0
19260/Edinburgh	4	4	6<br>1710	6<br>1800	7<br>1380	7<br>1510	7<br>1540	8<br>1440<br>2130	8<br>1440

Table S6: Meteorological data from DMI station 6011 Torshavn (Faroes)

Time → Met. Parameter ↓	0600	0700	0800	0900	1000	1100	1200
Cloud cover (%)	10	90	90	90	90	90	60
Relative humidity (%)	81	87	84	88	80	79	81
Precipitation last 10 min (mm)	0.0	0.3	0.3	0.3	0.0	0.0	0.0
Precipitation last 1 hr (mm)	0.3	0.5	0.5	0.5	0.0	0.0	0.0
MSLP (hPa)	1017.3	1017.7	1017.7	1018.2	1018.5	1019.0	1019.7
Global radiation (W m^{-2})	0.0	4.0	36.0	40.0	44.4	337.0	237.0
Global radiation mean of last 1 hr (W m^{-2})	0.0	1.0	27.0	72.0	32.0	248.0	261.0
Sun last 10 min (mins)	0.0	0.0	0.0	0.0	0.0	3.5	3.0
Sun last 1 hr (mins)	0.0	0.0	0.0	5.5	0.0	18.5	18.5
Temperature (°C)	6.7	5.7	6.4	6.5	7.2	8.1	7.9
Temp. max. last 1 hr	6.9	6.9	6.6	6.8	7.2	8.1	8.6
Temp. mean last 1 hr	6.6	6.4	6.2	6.6	6.7	7.7	8.0
Temp. min. last 1 hr	6.3	5.7	5.5	6.3	6.5	7.2	7.4
Wind direction (deg)	299	304	303	317	326	316	328
Wind speed (m s^{-1})	8.9	9.1	8.8	6.6	8.2	8.6	9.0

Table S7: Ten-minute meteorological data from DMI station 6010 Vaga Floghavn (Faroes)

Time	Cloud cover (%)	Relative humidity (%)	MSLP (hPa)	Temperature (°C)	Wind direction (deg)	Wind speed (m s^{-1})
0600	90	N/A	1017.9	5.9	291	7.2
0610	90	92	1017.9	5.9	297	8.8
0620	90	95	1017.9	5.8	295	7.7
0630	90	88	1018.0	5.9	293	8.2
0640	90	83	1018.0	5.9	298	9.3
0650	90	90	1018.0	5.7	294	8.2
0700	90	91	1018.0	5.6	289	8.8
0710	90	92	1018.1	5.6	291	8.8
0720	90	90	1018.1	5.8	293	7.7
0730	90	84	1017.9	6.1	297	8.8
0740	90	84	1018.0	6.1	304	10.3
0750	90	83	1018.0	6.3	302	8.2
0800	90	85	1018.1	6.2	299	8.8
0810	90	85	1018.3	6.2	295	8.8
0820	90	86	1018.3	6.3	299	8.2
0830	90	86	1018.3	6.2	300	7.7
0840	90	86	1018.4	6.4	315	8.8
0850	90	85	1018.5	6.8	315	10.8
0900	90	88	1018.6	6.3	316	11.3
0910	60	87	1018.8	6.4	319	11.8
0920	50	86	1018.9	6.3	320	12.4
0930	40	85	1019.1	6.2	320	11.3
0940	25	87	1019.2	5.9	320	10.8
0950	40	88	1019.5	5.9	322	10.3
1000	60	87	1019.6	5.8	326	9.8
1010	90	88	1019.7	5.8	325	9.3
1020	90	89	1020.0	5.7	322	10.3
1030	90	88	1020.1	5.7	324	9.8
1040	90	88	1020.0	6.0	315	10.3
1050	90	92	1020.1	6.1	314	11.3
1100	90	92	1020.1	6.1	310	11.3
1110	90	93	1020.2	6.2	312	11.8
1120	100	92	1020.4	6.1	315	12.9
1130	100	93	1020.6	6.1	312	11.8
1140	100	94	1020.8	5.7	317	12.4
1150	100	92	1020.9	5.8	331	10.8
1200	90	92	1020.9	5.7	333	11.3

Table S8: One-hour meteorological data from DMI stations: (a) 6009 Akraberg Fyr and (b) Kirkja (Fugloy)

(a) 6009 Akraberg Fyr

Time	Rel hum (%)	MSLP (hPa)	Temperature (°C)	Temp. max. last 1 hr	Temp. mean last 1 hr	Temp. min. last 1 hr	Wind direction (deg)	Wind speed (m s^{-1})
0600	98	1018.5	5.7	6.3	6.0	5.6	227	7.1
0700	93	1018.8	6.2	6.2	5.8	5.5	267	6.6
0800	91	1018.8	6.0	6.4	6.1	5.7	253	6.6
0900	87	1019.0	6.7	6.7	6.4	6.0	270	7.0
1000	92	1019.1	6.5	6.7	6.4	6.2	292	8.1
1100	82	1019.5	7.6	7.7	7.2	6.5	309	10.8
1200	84	1020.0	7.1	8.0	7.4	6.9	313	11.6

(b) Kirkja (Fugloy)

Time	MSLP (hPa)	Temperature (°C)	Temp. max. last 1 hr	Temp. mean last 1 hr	Temp. min. last 1 hr	Wind direction (deg)	Wind speed (m s^{-1})	Cloud cover (%)
0600	1016.1	6.0	6.5	6.3	5.9	298	10.9	100
0700	1016.2	6.1	6.1	5.9	5.8	299	9.0	90
0800	1016.7	6.0	6.2	5.9	5.4	313	7.5	90
0900	1017.1	6.4	6.4	5.8	5.5	305	6.6	90
1000	1017.8	6.3	6.6	6.2	6.1	312	6.8	90
1100	1018.4	6.6	7.0	6.5	6.0	307	7.6	90
1200	1020.5	5.8	7.5	6.6	5.6	107	4.0	90

Figure S1: Map showing positions of UK Met Office MMS weather stations used in this study.

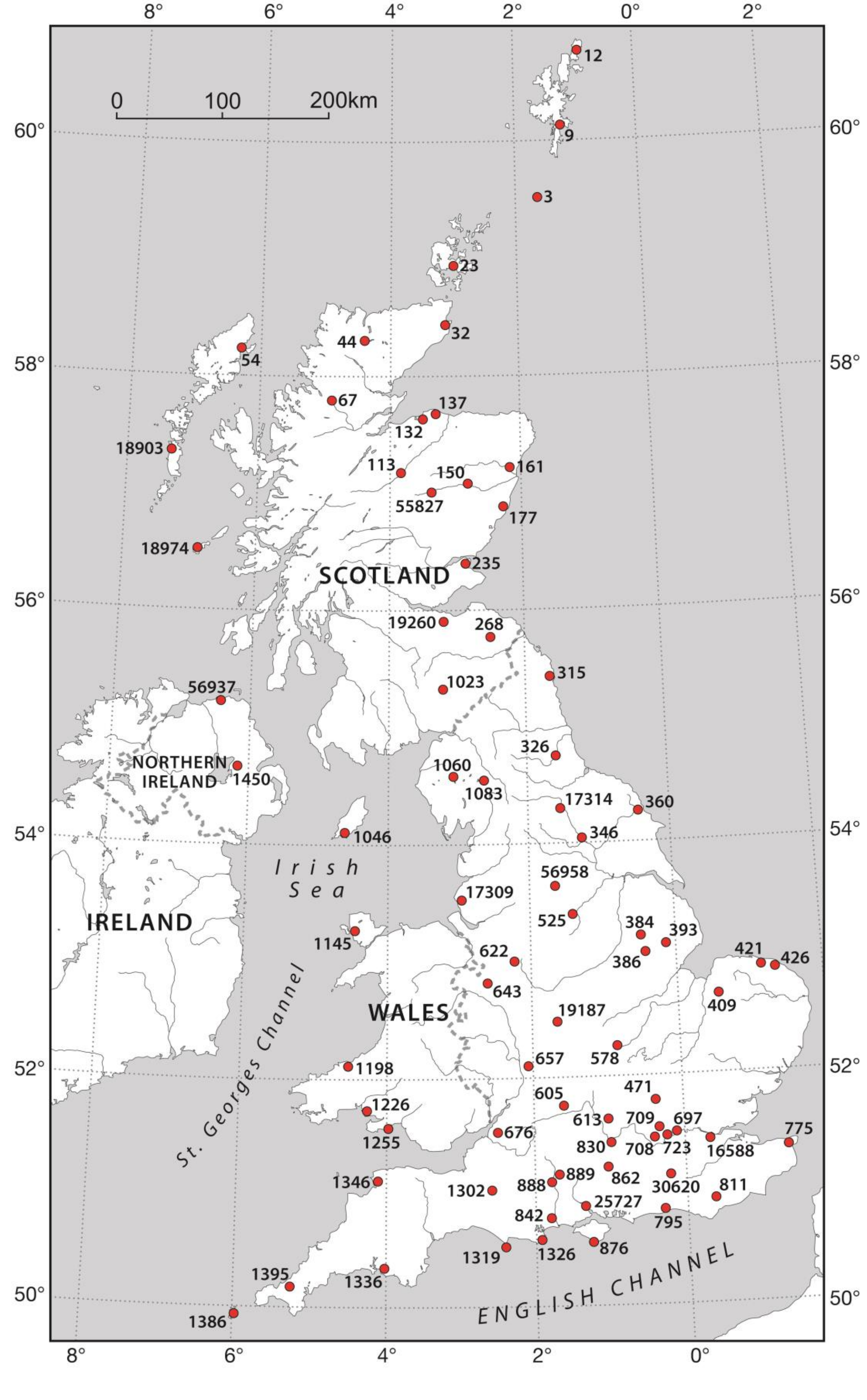

Figure S2: Map showing positions of Faroe Islands automatic weather stations used in this study.

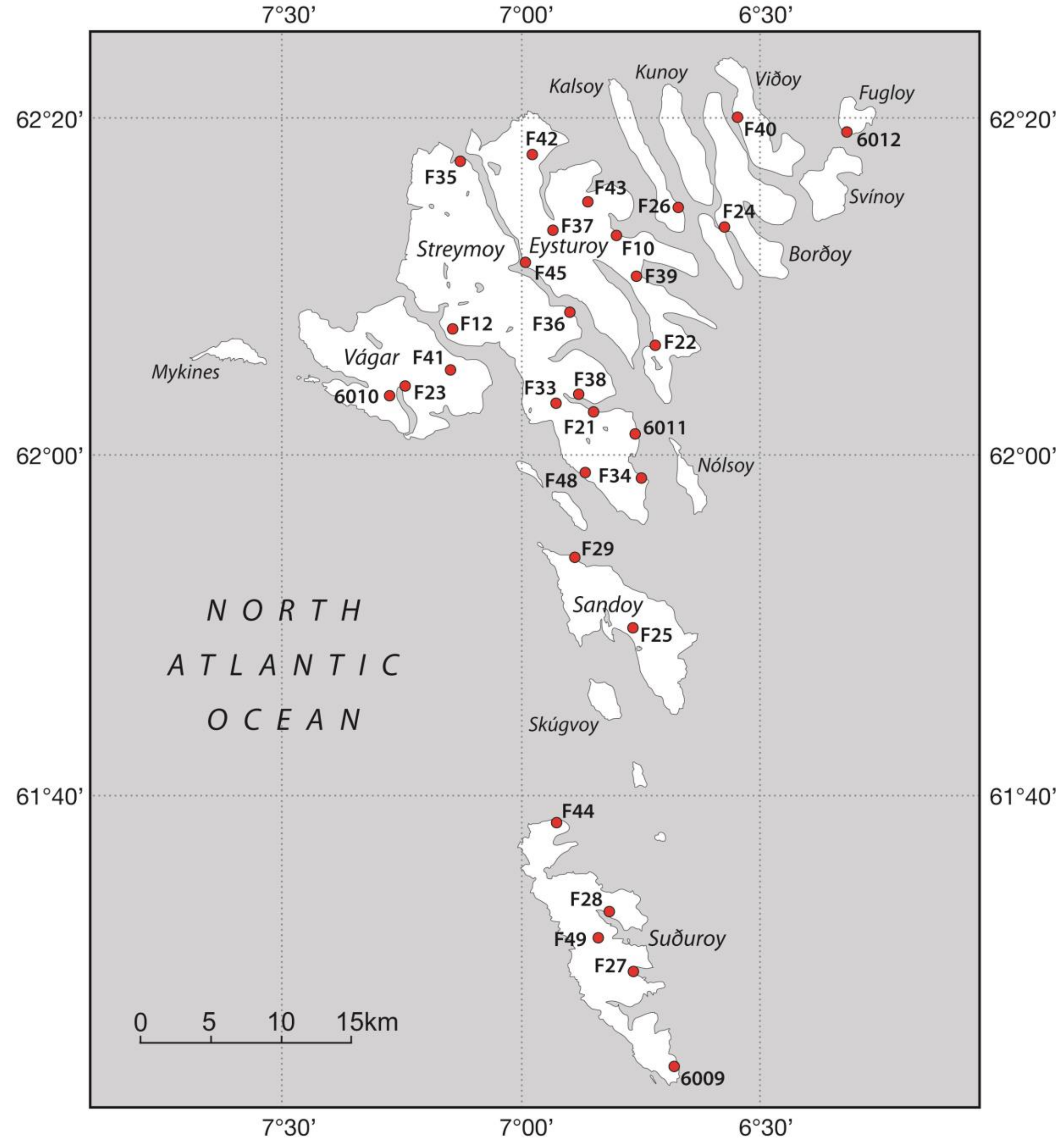

Figure S3: Map showing positions of Icelandic Met Office automatic weather stations measuring temperature used in this study.

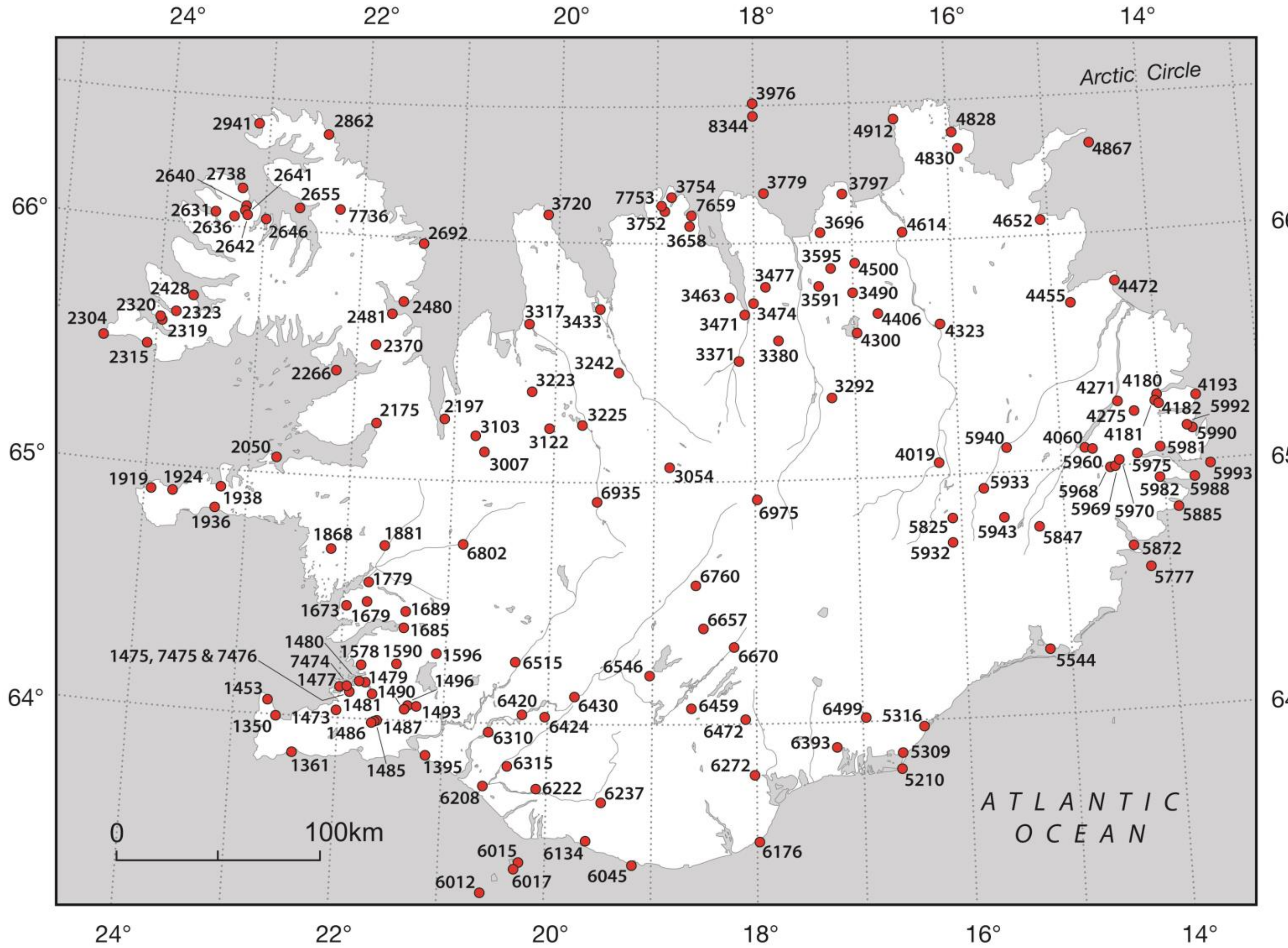

Figure S4: Photos of a typical automatic weather station (AWS) setup in the three different national regions: (a) UK Met Office Meteorological Monitoring System (MMS) AWS – the example shown is for Odiham in Hampshire; (b) Faroes (Landsverk) AWS – the example shown is F42 Gjáarskarð mountain pass; (c) Icelandic Meteorological Office (IMO) AWS at Árnes (station ID 6420) - typical thermometer setup at a rural automatic station in Iceland [photo credit Sigvaldi Árnason (2004)]. **Photo (d) shows EH's Davis Vantage Pro 2 AWS with Fan Assisted Radiation Shield at New Balderton, Newark-on-Trent, Nottinghamshire, UK.**

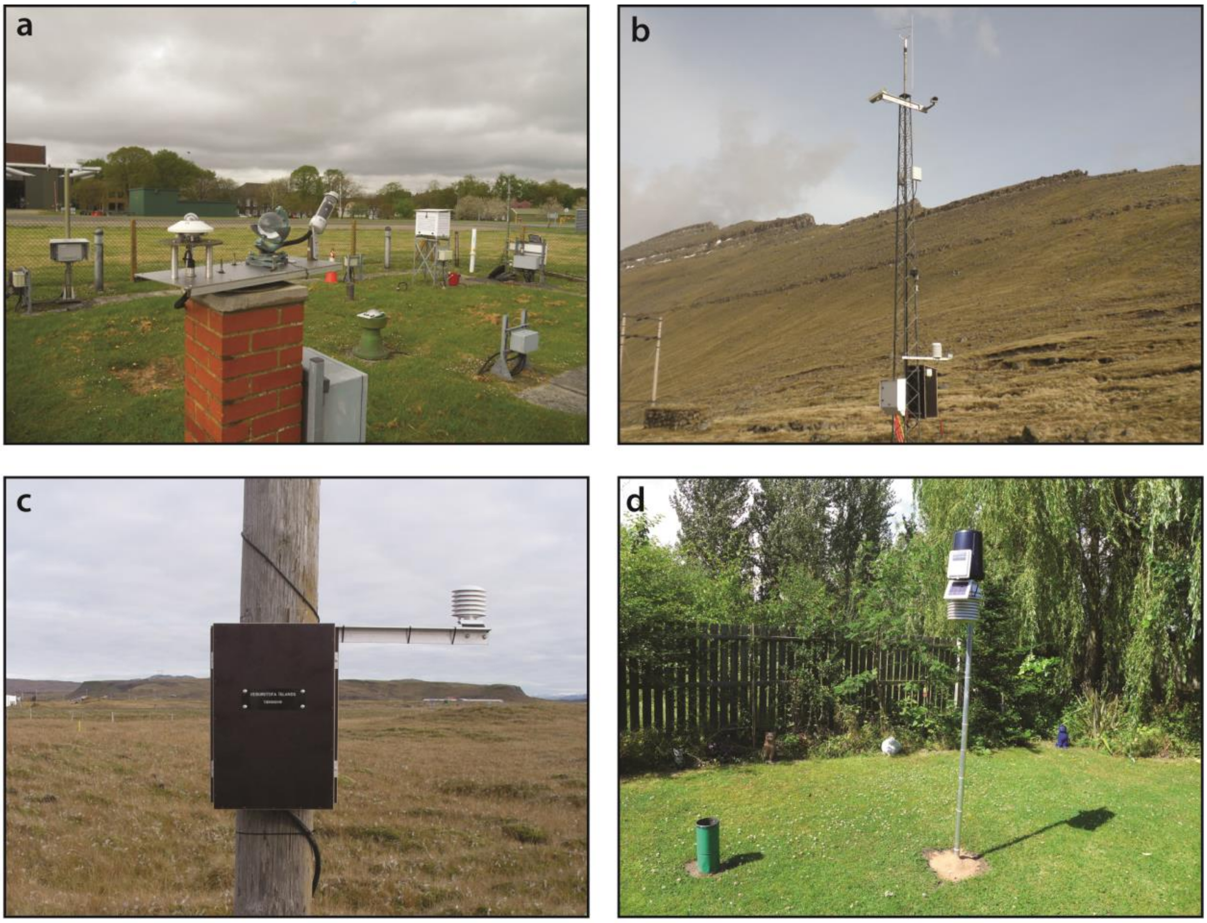

Figure S5: Wind direction at 63 weather stations across the UK between 6 and 12 UTC on 20 March 2015, with the bold red line marking the mean of all stations. The vertical lines C1, GE and C4 mark the times of first contact, maximum eclipse and fourth contact.

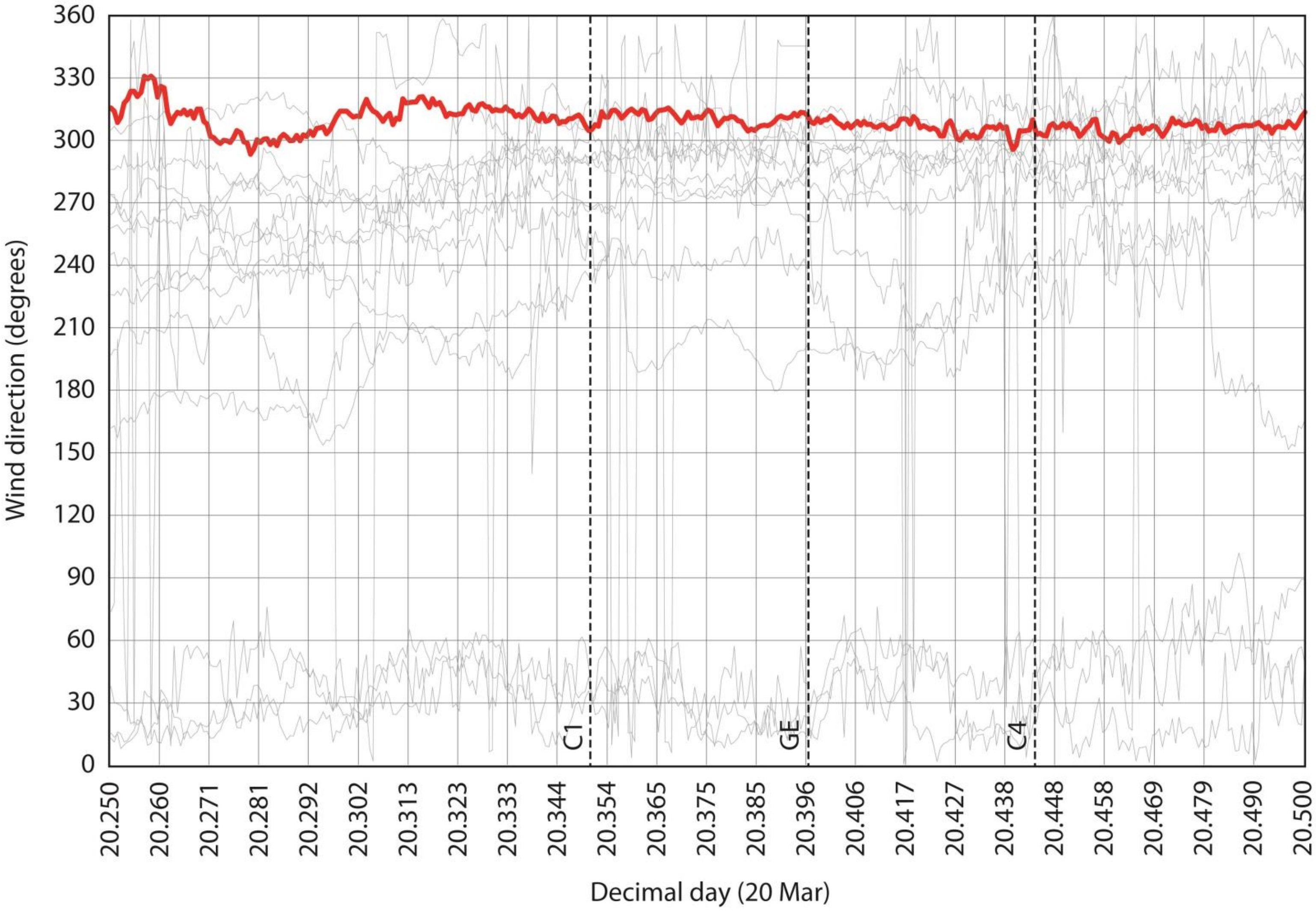

Figure S6: Mean sea-level pressure at 61 weather stations across the UK between 6 and 12 UTC on 20 March 2015, with the bold red line marking the mean of all stations.

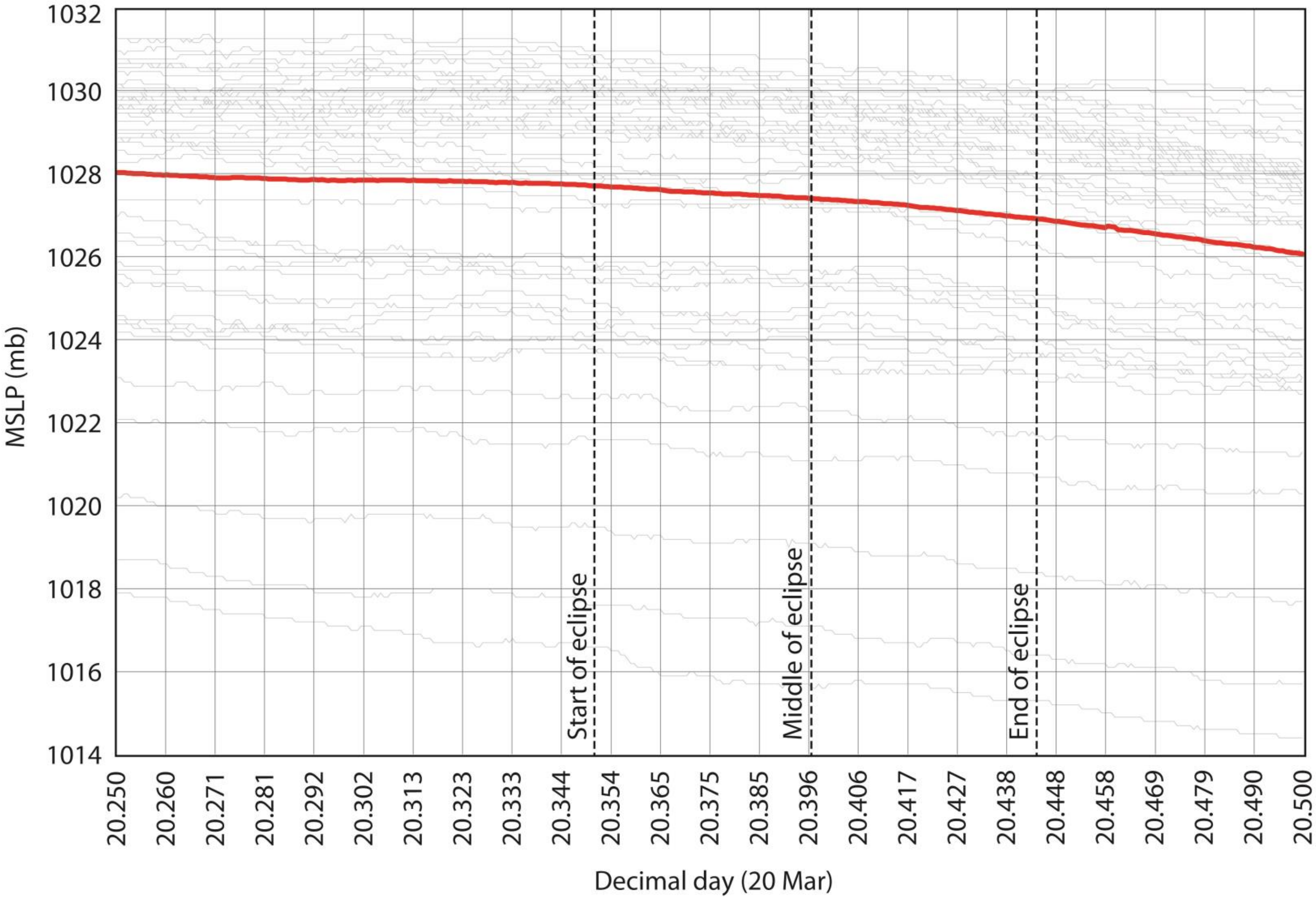

Figure S7: Mean-sea-level pressure (MSLP) values (hPa–1000x10), with 1 hPa isobars overplotted, at 09:30 on 20 March 2015, around the time of mid-eclipse over the UK.

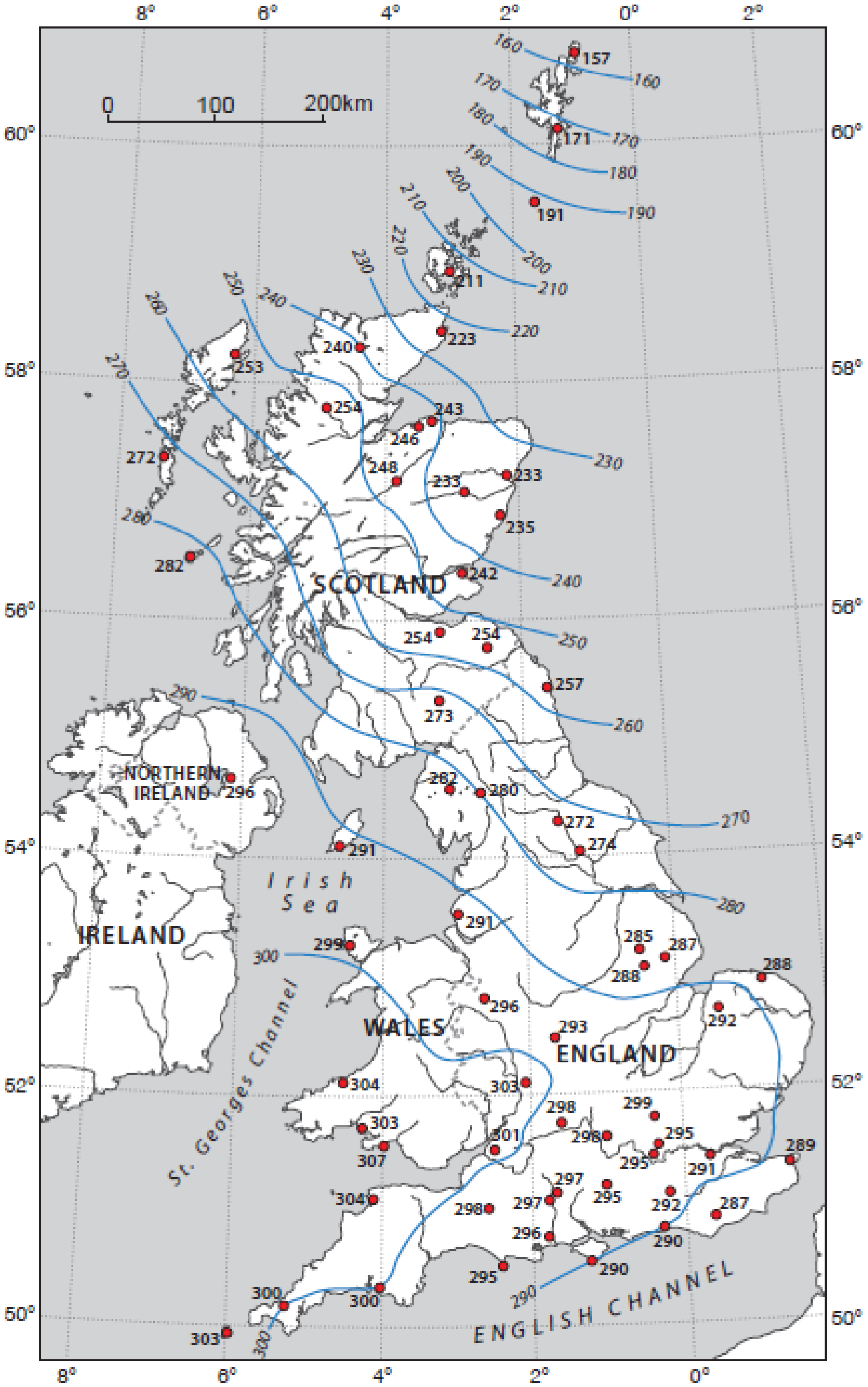

Figure S8. 10-min surface air temperature measured at Landsverk, Faroe Islands, automatic weather stations at elevations above sea-level of: (a) <100m and (b) ≥100 m on 20 March 2015. The vertical lines C1, GE and C4 mark the times of first contact, maximum eclipse and fourth contact.

(a)

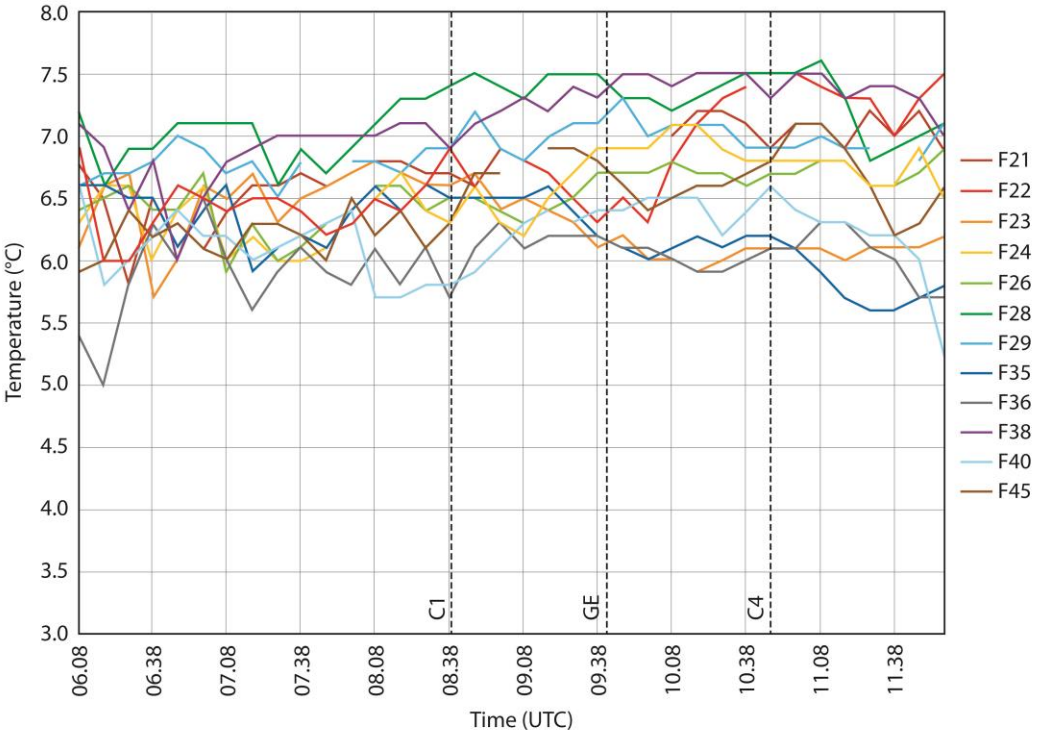

(b)

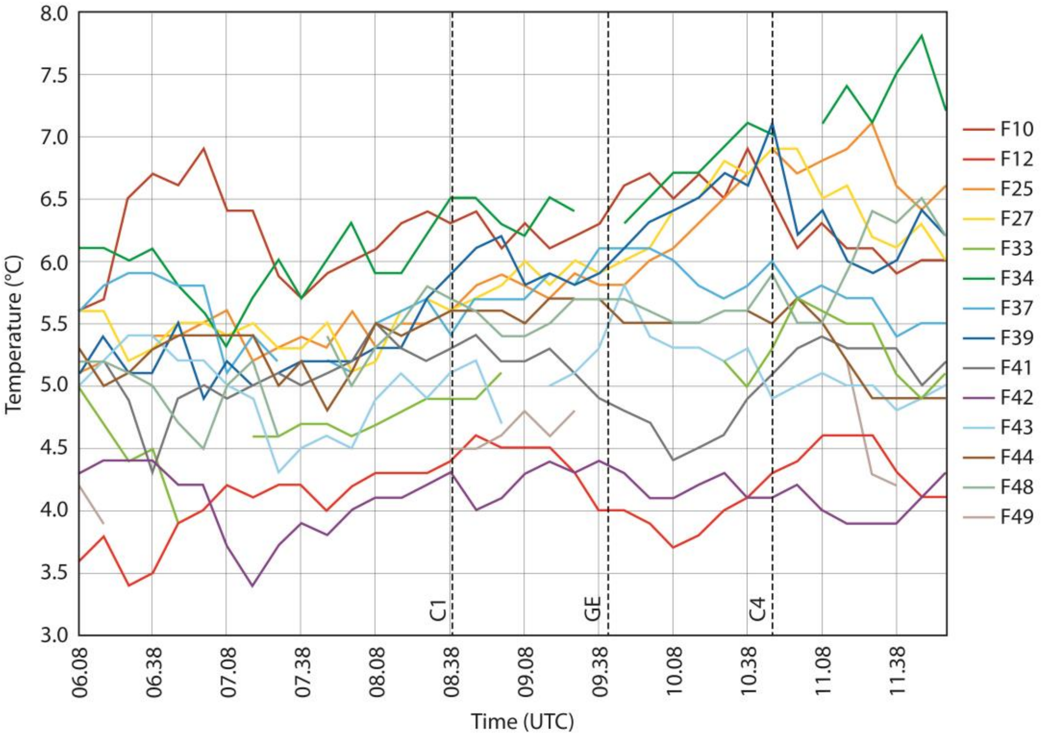

Figure S9. 10-min wind speed measured at Landsverk, Faroe Islands, automatic weather stations at elevations above sea-level of (a) <100m and (b) ≥100 m on 20 March 2015. The vertical lines C1, GE and C4 mark the times of first contact, maximum eclipse and fourth contact.

(a)

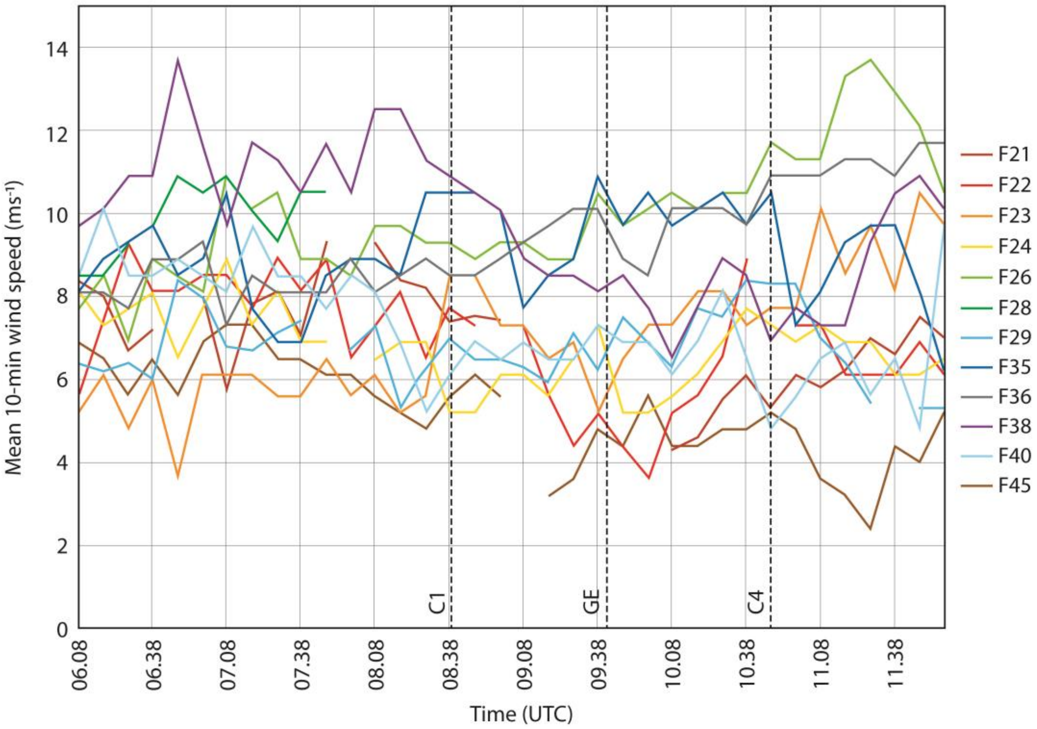

(b)

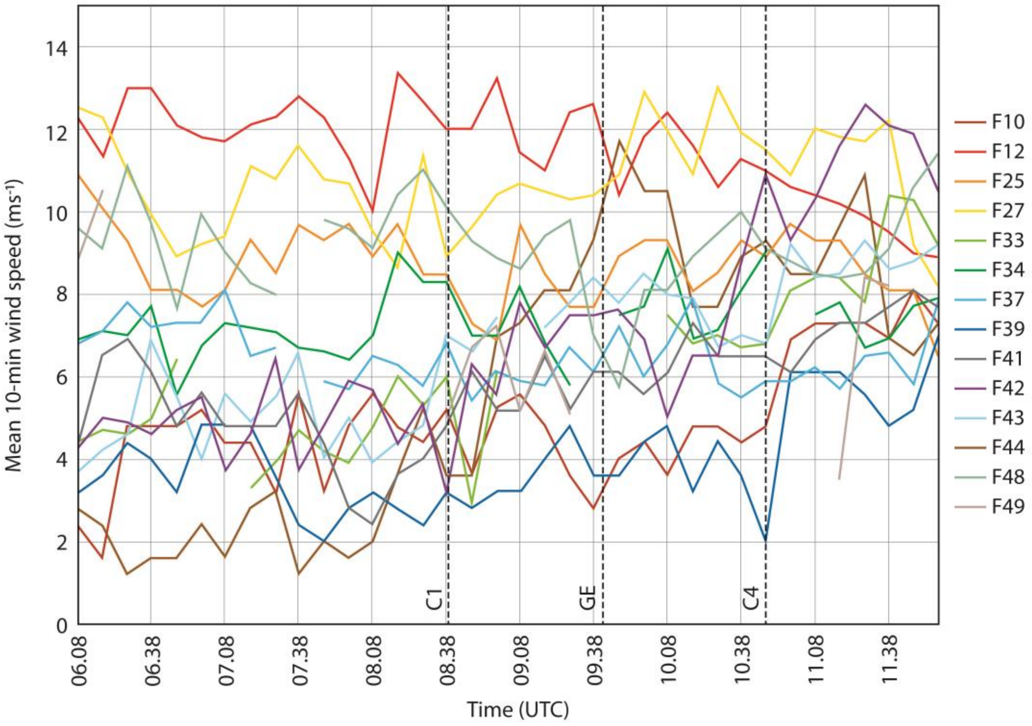

Figure S10. Station-level pressure measured at Landsverk, Faroe Islands, automatic weather stations at elevations above sea-level of (a) <100m and (b) ≥100 m on 20 March 2015. The vertical lines C1, GE and C4 mark the times of first contact, maximum eclipse and fourth contact.

(a)

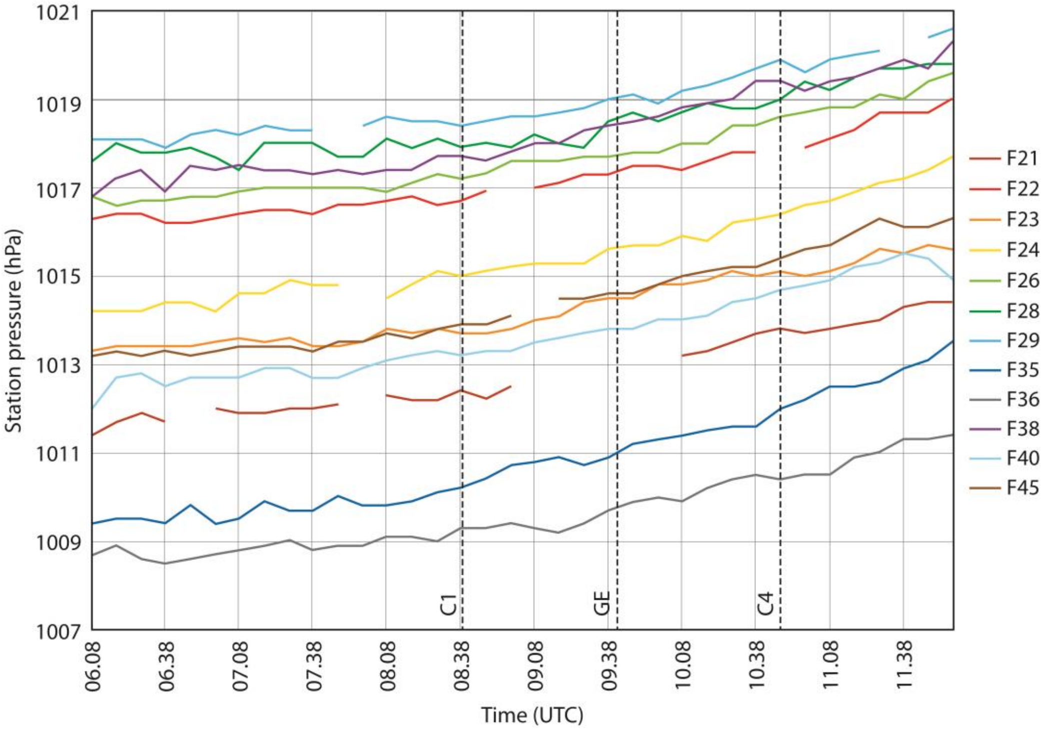

(b)

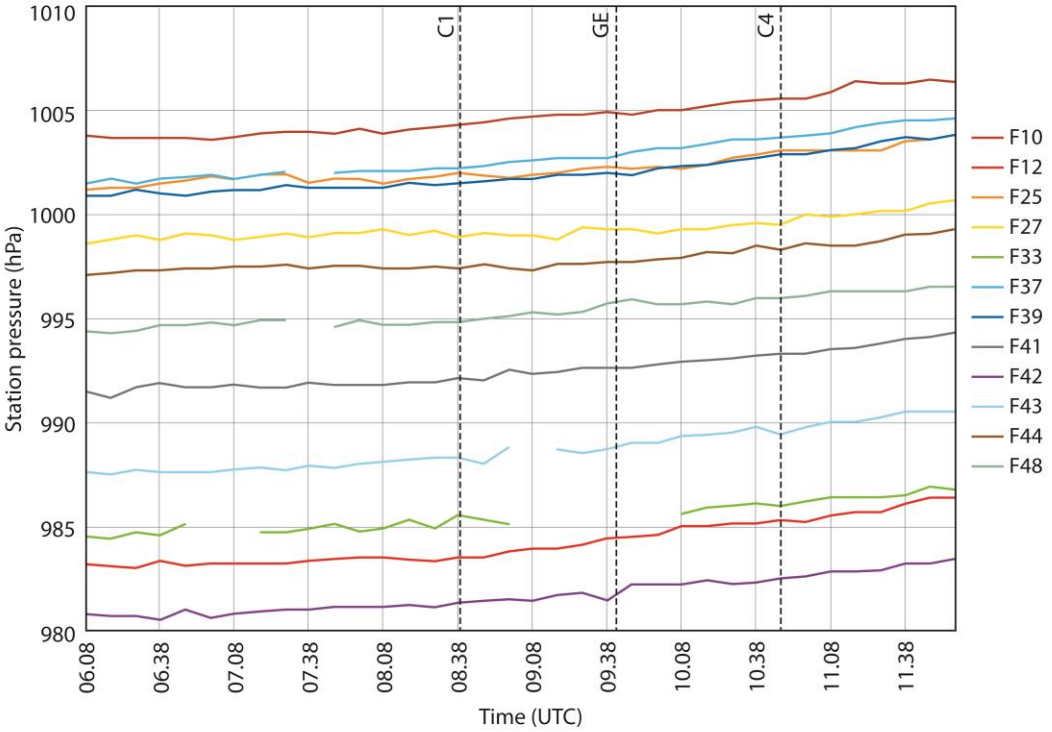

Figure S11: (a) Mean sea-level pressure (MSLP) values (hPa-1000x10) at Icelandic Met Office (IMO) automatic weather stations (AWSs) at 09:40 UTC (around the time of mid-eclipse) on 20 March 2015, with the blue contours depicting 1 hPa isobars; (b) MSLP changes (hPa) at IMO AWSs during the first and second halves of the eclipse (08:40-09:40 and 09:40-10:40).

(a)

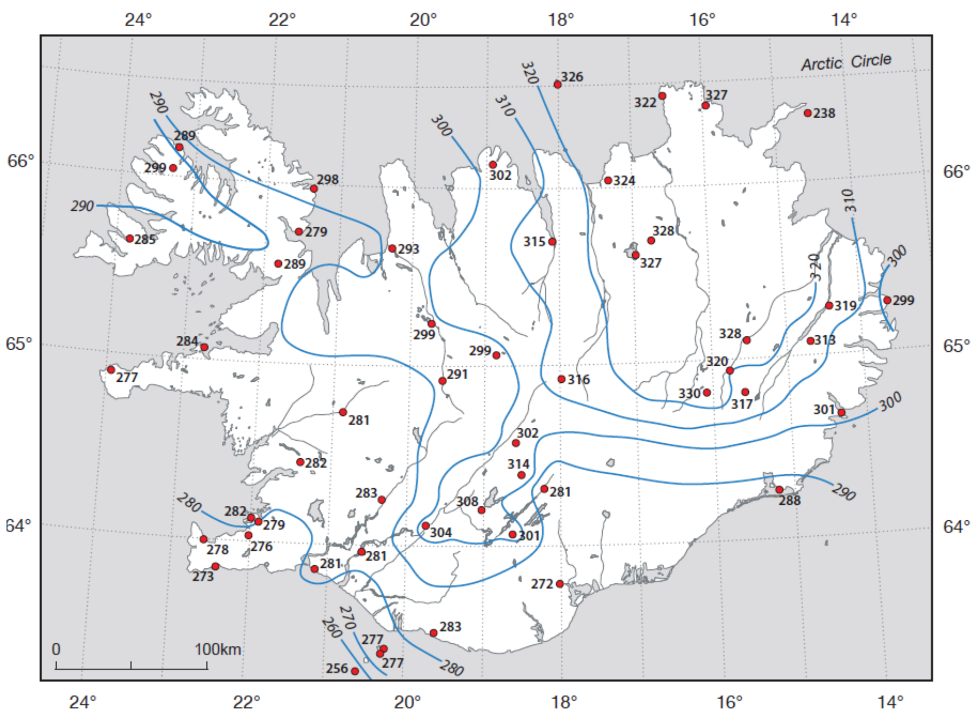

(b)

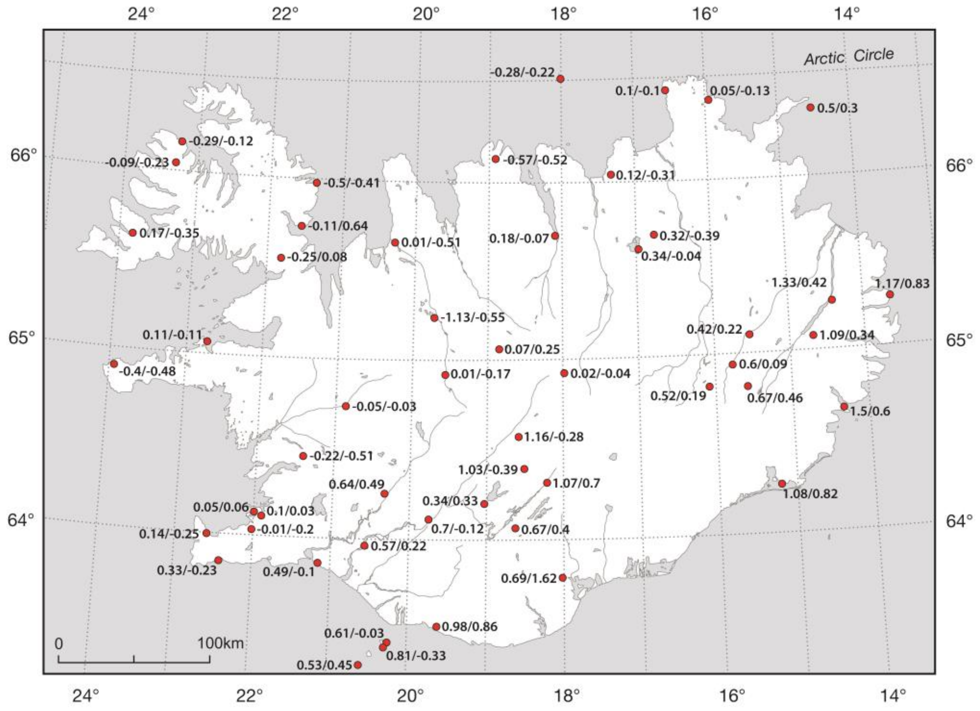

Supplement: Supplement Tables and Figures [file rsta20150212supp1.pdf]
